# Joint analysis of hemoglobin-to-RDW and creatinine-to-albumin ratios for mortality prediction in critical heart failure

**DOI:** 10.1016/j.isci.2026.115740

**Published:** 2026-04-17

**Authors:** Shengzhang Chen, Fei Wang, Binyan Chen, Qian Lu, Miwen Zou, Jiaying Lou, Fuman Cai, Pan Huang, Jianghua Zhou, Haodi Dang

**Affiliations:** 1 Department of Cardiology, The First Affiliated Hospital of Wenzhou Medical University, Wenzhou, China; 2 School of Nursing, Wenzhou Medical University, Wenzhou, China

**Keywords:** health sciences, medicine, medical specialty, internal medicine, cardiovascular medicine

## Abstract

Hemoglobin-to-red cell distribution width ratio (HRR) and creatinine-to-albumin ratio (CAR) are simple and easily obtainable biomarkers reflecting anemia/inflammation and renal and nutritional status, respectively. We investigated whether these markers predict mortality in intensive care unit (ICU) patients with heart failure (HF). In a retrospective analysis of 31,454 ICU cases from three critical care databases (MIMIC-IV, MIMIC-III, eICU-CRD) and an independent hospital cohort, Cox regression models assessed associations with 28-day all-cause mortality (primary outcome) and 90-day, 180-day, and 1-year mortality. Higher HRR was independently associated with lower 28-day mortality, whereas higher CAR predicted higher 28-day mortality risk. These associations remained consistent at 90-day, 180-day, and 1-year follow-up. Patients with combined low HRR and high CAR had the greatest 28-day mortality risk. HRR and CAR thus serve as independent predictors of short-term mortality in ICU patients with HF, and their joint assessment may improve early risk stratification and management.

## Highlights

• HRR and CAR independently predict mortality across four ICU cohorts and time points
• Joint HRR-CAR assessment outperforms single markers and traditional severity scores
• Low HRR with high CAR identifies the highest-risk phenotype with robust external validation

## Graphical abstract

**Figure:**
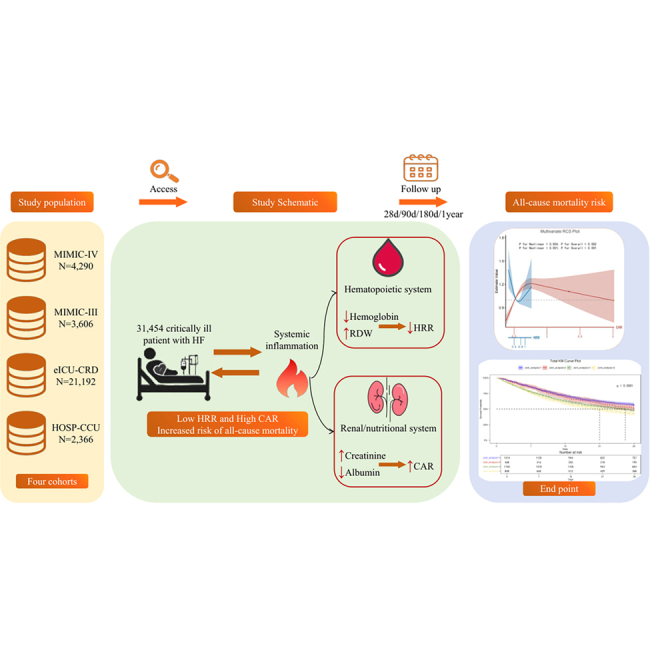

## Introduction

Heart failure (HF) is a clinical syndrome characterized by the presence of signs and symptoms resulting from structural or functional cardiac abnormalities, which can be confirmed through objective evidence, including elevated levels of natriuretic peptides or clear evidence of pulmonary/corporeal circulation stasis.^1^ The lifetime risk of HF at age 45 years ranges from 20% to 46%, with higher risks in the elderly, hypertensive, and hospitalized multimorbid patients.^2^ The prevalence of HF varies from study to study due to differences in definitions and analyses, but the temporal trend is consistent across reports and suggests an increasing prevalence of HF.^3^ HF affects approximately 6.5 million adults in the United States.^4^ While Asia, the fastest-growing and most populous region in the world, accounts for 60% of the global burden of cardiovascular disease. The HF situation is even more critical in Asia in the context of an aging population, often presenting at younger ages and with greater severity of presentation.^5^^,^^6^^,^^7^^,^^8^ The time duration of patients with HF in intensive care unit (ICUs) is longer, accounting for 33% of total ICU patient days, with an increasing burden and demonstrating the highest risk, most complex, and resource-intensive disease states.^9^^,^^10^ Given the growing prevalence of HF, primary prevention of HF needs to be prioritized, and a concerted effort needs to be made to mitigate the persistent and pervasive problems in the development of HF.

There are numerous pathophysiological factors that contribute to HF, and inflammation unarguably occupies an important part, not only as a hallmark feature but also as an active driver of disease progression.^11^ Immediately after myocardial injury, the immune system is activated, and the inflammatory response at this point is a cytoprotective program initiated by the body to restore homeostasis. However, if inflammation is prolonged or imbalanced, it can turn from repair to destruction, resulting in ongoing myocardial injury and pathological remodeling.^12^^,^^13^ Early recognition of the inflammatory response and status of the patient is extremely important for patients with HF. Hemoglobin-to-red cell distribution width ratio (HRR) is a novel composite inflammatory biomarker proposed by Sun et al. in 2016,^14^ and studies have demonstrated that lower levels of HRR are associated with a number of adverse outcomes and are involved in the onset, progression, and prognosis of a wide range of diseases.^15^^,^^16^ Creatinine-to-albumin ratio (CAR) has been mentioned by Zhao et al.^17^ Serum creatinine (Cr) is most commonly used for the assessment of renal function, but some studies have also confirmed that Cr has a close association with cardiovascular aspects and can be used for the prediction of related outcomes in cardiovascular events, including HF.^18^^,^^19^ CAR is a practical, consistent, and easily measurable biochemical tool, and it can even be used to assess adverse outcomes in critically ill patients.^20^

Since HF is an incurable, life-threatening disease associated with poor survival, increasing prevalence, and a significant social and economic burden on patients, caregivers, and the healthcare system, primary prevention has outstanding public health value. Although previous studies for HRR and CAR have been shown to predict prognosis in relevant patients.^17^^,^^21^ A single indicator may only reflect a single dimension of inflammation or immunity, resulting in limited predictive efficacy. However, integrating HRR and CAR to incorporate three-dimensional information on immunity, inflammation, and nutrition can significantly improve the accuracy of risk stratification and simplify clinical decision-making. Therefore, this study aimed to investigate the value of HRR and CAR in predicting mortality in such patients using four large databases (MIMIC-III, MIMIC-IV, eICU, HOSP-CCU validation cohort).

## Results

### Description of baseline characteristics

**Figure 1:**
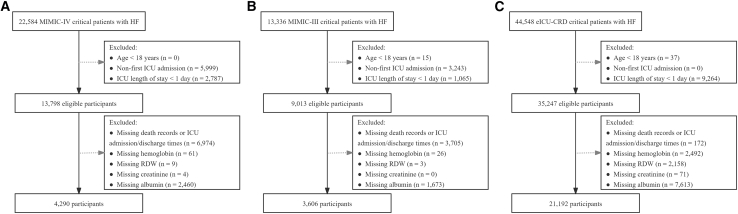
Flowchart of patient selection from three publicly available databases for critically ill patients with heart failure Notes: (A) MIMIC-IV database, (B) MIMIC-III database, and (C) eICU-CRD database. Abbreviations: HF, heart failure; ICU, intensive care unit; MIMIC-IV/MIMIC-III, Medical Information Mart for Intensive Care IV/III; eICU-CRD, eICU Collaborative Research Database; RDW, red cell distribution width.

This study systematically evaluated the value of HRR and CAR in the risk stratification of patients with HF using three major public critical care databases: MIMIC-IV, MIMIC-III, and eICU-CRD (Figure 1). The results showed that the baseline characteristics of patients in MIMIC-IV and MIMIC-III were similar, with a median age of 77 years and male proportions of 55.41% and 53.91%, respectively. In contrast, patients in the eICU-CRD database were relatively younger, with a median age of 67 years and 53.35% male (Table 1).

**Table 1:** Baseline characteristics of study participants from the MIMIC-IV, MIMIC-III, and eICU databases

Variable	MIMIC-IV	MIMIC-III	eICU
	*N* = 4,290	*N* = 3,606	*N* = 21,191
Age, Median (Q1 - Q3), years	77.00 (68.00–85.00)	77.00 (68.00–84.00)	67.00 (55.00–78.00)
Alb, Median (Q1 - Q3), g/dL	3.00 (2.50–3.40)	3.00 (2.60–3.50)	2.90 (2.40–3.30)
ALT, Median (Q1 - Q3), IU/L	25.00 (14.00–55.00)	24.00 (15.00–49.00)	35.00 (20.00–75.00)
Anoin gap, Median (Q1 - Q3), mEq/L	15.00 (13.00–18.00)	15.00 (13.00–17.00)	13.00 (10.00–16.70)
AST, Median (Q1 - Q3), IU/L	39.00 (23.00–87.00)	33.00 (22.00–65.00)	42.00 (24.00–99.00)
Bilirubin total, Median (Q1 - Q3), mg/dL	0.70 (0.40–1.20)	0.60 (0.40–1.10)	0.80 (0.50–1.40)
BUN, Median (Q1 - Q3), mg/dL	35.00 (22.00–56.00)	31.00 (20.00–49.00)	35.00 (21.00–57.00)
Calcium total, Median (Q1 - Q3), mg/dL	8.40 (7.90–8.90)	8.40 (7.90–8.90)	8.80 (8.30–9.20)
CAR, Median (Q1 - Q3)	0.53 (0.34–0.91)	0.44 (0.30–0.75)	0.52 (0.32–0.97)
Chloride, Median (Q1 - Q3), mEq/L	101.00 (97.00–106.00)	103.00 (99.00–107.00)	109.00 (104.00–113.00)
Cr, Median (Q1 - Q3), mg/dL	1.50 (1.00–2.60)	1.30 (0.90–2.20)	1.43 (0.92–2.60)
Glu, Median (Q1 - Q3), mg/dL	133.00 (106.00–177.00)	126.00 (101.00–168.00)	201.00 (153.00–276.00)
Hb, Median (Q1 - Q3), g/dL	9.80 (8.40–11.40)	10.60 (9.40–11.90)	11.40 (10.10–12.90)
Hematocrit, Median (Q1 - Q3), %	30.70 (26.30–35.40)	32.00 (28.50–35.80)	34.80 (31.00–39.00)
HRR, Median (Q1 - Q3)	0.60 (0.49–0.75)	0.68 (0.57–0.81)	0.71 (0.58–0.86)
Los hosp day, Median (Q1 - Q3)	11.58 (6.57–19.74)	11.83 (7.19–19.84)	8.37 (4.98–14.34)
Los icu day, Median (Q1 - Q3)	3.97 (2.15–8.03)	4.34 (2.28–9.17)	3.50 (2.09–6.68)
Platelet count, Median (Q1 - Q3), K/uL	187.00 (130.00–259.00)	209.00 (148.00–281.00)	242.00 (174.00–333.00)
Potassium, Median (Q1 - Q3), mEq/L	4.30 (3.80–4.80)	4.20 (3.80–4.60)	4.60 (4.20–5.10)
RBC, Median (Q1 - Q3), m/uL	3.33 (2.84–3.89)	3.54 (3.11–4.00)	3.85 (3.40–4.34)
RDW, Median (Q1 - Q3), %	16.00 (14.60–17.90)	15.40 (14.20–17.10)	15.80 (14.50–17.80)
SAPSII, Median (Q1 - Q3)	44.00 (36.00–53.00)	43.00 (36.00–52.00)	37.00 (28.00–49.00)
Sodium, Median (Q1 - Q3), mEq/L	138.00 (134.00–141.00)	139.00 (136.00–141.00)	142.00 (139.00–146.00)
SOFA, Median (Q1 - Q3)	6.00 (4.00–9.00)	5.00 (3.00–8.00)	5.00 (3.00–8.00)
WBC, Median (Q1 - Q3), K/uL	11.20 (7.80–16.20)	10.30 (7.30–14.50)	14.80 (10.50–20.70)
Weight, Median (Q1 - Q3), kg	76.65 (64.00–92.80)	74.25 (62.10–88.60)	79.60 (65.80–98.00)

**Gender, %**			

Female	1,913 (44.59%)	1,662 (46.09%)	9,886 (46.65%)
Male	2,377 (55.41%)	1,944 (53.91%)	11,305 (53.35%)

**Ventilation, %**			

No	554 (12.91%)	1,816 (50.36%)	12,470 (58.85%)
Yes	3,736 (87.09%)	1,790 (49.64%)	8,721 (41.15%)

**GCS, %**			

3–8	371 (8.65%)	278 (7.71%)	5,606 (26.45%)
9–12	412 (9.60%)	324 (8.99%)	3,873 (18.28%)
13–15	3,507 (81.75%)	3,004 (83.31%)	11,712 (55.27%)

**HRR Group, %**			

Tertiles 1	1,430 (33.33%)	1,202 (33.33%)	7,065 (33.34%)
Tertiles 2	1,430 (33.33%)	1,203 (33.36%)	7,062 (33.33%)
Tertiles 3	1,430 (33.33%)	1,201 (33.31%)	7,064 (33.33%)

**Joint analysis, %**			

HRR Low and CAR Low	883 (20.58%)	709 (19.66%)	4,007 (18.91%)
HRR High and CAR Low	1,258 (29.32%)	1,074 (29.78%)	6,593 (31.11%)
HRR Low and CAR High	1,255 (29.25%)	1,092 (30.28%)	6,585 (31.07%)
HRR High and CAR High	894 (20.84%)	731 (20.27%)	4,006 (18.90%)

**CAR Group, %**			

Tertiles 1	1,467 (34.20%)	1,207 (33.47%)	7,071 (33.37%)
Tertiles 2	1,405 (32.75%)	1,204 (33.39%)	7,058 (33.31%)
Tertiles 3	1,418 (33.05%)	1,195 (33.14%)	7,062 (33.33%)

**Insulin, %**			

No	1,626 (37.90%)	1,051 (29.15%)	15,151 (71.50%)
Yes	2,664 (62.10%)	2,555 (70.85%)	6,040 (28.50%)

**Beta blocker, %**			

No	1,406 (32.77%)	991 (27.48%)	12,974 (61.22%)
Yes	2,884 (67.23%)	2,615 (72.52%)	8,217 (38.78%)

**Neuromuscular blocker, %**			

No	4,111 (95.83%)	3,589 (99.53%)	20,636 (97.38%)
Yes	179 (4.17%)	17 (0.47%)	555 (2.62%)

**Sedative analgesic, %**			

No	1,715 (39.98%)	1,557 (43.18%)	12,960 (61.16%)
Yes	2,575 (60.02%)	2,049 (56.82%)	8,231 (38.84%)

**Vasopressin, %**			

No	1,495 (34.85%)	1,820 (50.47%)	15,817 (74.64%)
Yes	2,795 (65.15%)	1,786 (49.53%)	5,374 (25.36%)

**Glucocorticoid, %**			

No	2,768 (64.52%)	2,635 (73.07%)	17,321 (81.74%)
Yes	1,522 (35.48%)	971 (26.93%)	3,870 (18.26%)

**ACEI, %**			

No	3,481 (81.14%)	2,514 (69.72%)	20,732 (97.83%)
Yes	809 (18.86%)	1,092 (30.28%)	459 (2.17%)

**ARB, %**			

No	3,958 (92.26%)	3,379 (93.70%)	21,096 (99.55%)
Yes	332 (7.74%)	227 (6.30%)	95 (0.45%)

**CB, %**			

No	3,543 (82.59%)	3,411 (94.59%)	21,093 (99.54%)
Yes	747 (17.41%)	195 (5.41%)	98 (0.46%)

**MI, %**			

No	3,543 (82.59%)	3,388 (93.95%)	18,321 (86.46%)
Yes	747 (17.41%)	218 (6.05%)	2,870 (13.54%)

**HLD, %**			

No	2,392 (55.76%)	3,162 (87.69%)	20,384 (96.19%)
Yes	1,898 (44.24%)	444 (12.31%)	807 (3.81%)

**T2DM, %**			

No	2,456 (57.25%)	2,457 (68.14%)	20,323 (95.90%)
Yes	1,834 (42.75%)	1,149 (31.86%)	868 (4.10%)

**CA, %**			

No	3,431 (79.98%)	3,112 (86.30%)	20,112 (94.91%)
Yes	859 (20.02%)	494 (13.70%)	1,079 (5.09%)

**CKD, %**			

No	2,450 (57.11%)	2,993 (83.00%)	13,105 (61.84%)
Yes	1,840 (42.89%)	613 (17.00%)	8,086 (38.16%)

**CVA, %**			

No	3,787 (88.28%)	3,350 (92.90%)	19,744 (93.17%)
Yes	503 (11.72%)	256 (7.10%)	1,447 (6.83%)

**PNA, %**			

No	2,545 (59.32%)	1,970 (54.63%)	8,551 (40.35%)
Yes	1,745 (40.68%)	1,636 (45.37%)	12,640 (59.65%)

**HEP, %**			

No	4,152 (96.78%)	3,577 (99.20%)	20,763 (97.98%)
Yes	138 (3.22%)	29 (0.80%)	428 (2.02%)

**LC, %**			

No	3,917 (91.31%)	3,405 (94.43%)	19,077 (90.02%)
Yes	373 (8.69%)	201 (5.57%)	2,114 (9.98%)

**AKI, %**			

No	1,580 (36.83%)	1,899 (52.66%)	10,968 (51.76%)
Yes	2,710 (63.17%)	1,707 (47.34%)	10,223 (48.24%)

**HTN, %**			

No	3,504 (81.68%)	2,456 (68.11%)	16,584 (78.26%)
Yes	786 (18.32%)	1,150 (31.89%)	4,607 (21.74%)

**28-Day ICU Survival Status, %**			

No	2,118 (49.37%)	2,291 (63.53%)	18,614 (87.84%)
Yes	2,172 (50.63%)	1,315 (36.47%)	2,577 (12.16%)

**90-Day ICU Survival Status, %**			

No	1,315 (30.65%)	1,678 (46.53%)	18,557 (87.57%)
Yes	2,975 (69.35%)	1,928 (53.47%)	2,634 (12.43%)

**180-Day ICU Survival Status, %**			

No	914 (21.31%)	1,309 (36.30%)	18,555 (87.56%)
Yes	3,376 (78.69%)	2,297 (63.70%)	2,636 (12.44%)

**1-Year ICU Survival Status, %**			

No	454 (10.58%)	972 (26.96%)	18,555 (87.56%)
Yes	3,836 (89.42%)	2,634 (73.04%)	2,636 (12.44%)

**ICU Mortality, %**			

No	2,704 (63.03%)	2,511 (69.63%)	18,555 (87.56%)
Yes	1,586 (36.97%)	1,095 (30.37%)	2,636 (12.44%)

Abbreviations: Q1, first quartile; Q3, third quartile; Alb, albumin; ALT, alanine aminotransferase; AST, aspartate aminotransferase; BUN, blood urea nitrogen; CAR, creatinine-to-albumin ratio; Cr, creatinine; Glu, glucose; Hb, hemoglobin; HRR, hemoglobin to red cell distribution width ratio; Los, length of stay; RBC, red blood cell count; RDW, red cell distribution width; SAPSII, Simplified Acute Physiology Score II; SOFA, Sequential Organ Failure Assessment; WBC, white blood cell count; GCS, Glasgow Coma Scale; QBin, quartile classification; ACEI, angiotensin-converting enzyme inhibitors; ARB, angiotensin receptor blockers; CB, chronic bronchitis; MI, myocardial infarction; HLD, hyperlipidemia; T2DM, type 2 diabetes mellitus; CA, cancer; CKD, chronic kidney disease; CVA, cerebrovascular accident; PNA, pneumonia; HEP, hepatitis; LC, liver cirrhosis; AKI, acute kidney injury; HTN, hypertension.

In MIMIC-IV, patients in the low HRR group exhibited lower hemoglobin and albumin levels, along with higher RDW, BUN, and Cr. The high CAR group showed higher Cr, BUN, and lower albumin. This group also had higher ICU mortality, lower 28-day to 1-year survival rates, higher incidence of AKI and CKD, and higher SOFA and SAPSII scores, indicating more severe illness (Table S1). Data from MIMIC-III further validated these trends: The high CAR group had significantly elevated Cr, the highest SOFA and SAPSII scores, significantly higher prevalence of AKI and CKD, more frequent use of vasopressors and insulin, and the lowest 28-day survival (Table S2). The large-sample analysis from eICU-CRD reinforced these conclusions. The low HRR group had the lowest hemoglobin and albumin levels, while the high CAR group had the highest Cr and the highest incidence of AKI and CKD, along with higher rates of mechanical ventilation and vasopressor use (Table S3). These findings were consistently replicated in the independent HOSP-CCU validation cohort. Patients with combined low HRR and high CAR exhibited the most unfavorable profile, characterized by the lowest hemoglobin (8.20 g/dL) and albumin (3.02 g/dL), highest Cr (3.24 mg/dL), longest ICU stay (14 days), and highest burden of comorbidities including T2DM (54.19%), hypertension (77.83%), and CKD (85.22%) (Table S4). This independent validation confirms that the HRR-CAR combination robustly identifies high-risk HF patients with poor prognosis in real-world clinical settings.

### Association of HRR and CAR with different clinical outcomes

In the MIMIC-IV cohort, HRR was significantly associated with reduced 28-day mortality (per 1-unit increase: HR = 0.23, 95% CI: 0.15–0.35), while CAR was associated with increased risk (per 1-unit increase: HR = 1.14, 95% CI: 1.08–1.21) (Table 2). In MIMIC-III, HRR showed an even stronger protective effect (HR = 0.14, 95% CI: 0.08–0.24), whereas CAR’s effect remained moderate. Conversely, in the eICU-CRD cohort, HRR lost significance in the fully adjusted model, while CAR demonstrated stronger predictive value for mortality risk.

**Table 2:** Association of HRR and CAR with 28-day mortality in three critical ill cohorts

Cohort	Exposure	Level	Model 1 HR (95% CI)	*p* value	Model 2 HR (95% CI)	*p* value	Model 3 HR (95% CI)	*p* value
MIMIC-IV	HRR	continuous	0.78 (0.62,0.98)	0.033	0.76 (0.60,0.96)	0.021	0.23 (0.15,0.35)	<0.001
		T1	Ref.	–	Ref.	–	Ref.	–
		T2	0.88 (0.80,0.98)	0.018	0.88 (0.79,0.97)	0.011	0.79 (0.70,0.88)	<0.001
		T3	0.89 (0.80,0.98)	0.02	0.88 (0.79,0.97)	0.011	0.63 (0.54,0.74)	<0.001
	CAR	continuous	1.11 (1.06,1.17)	<0.001	1.13 (1.07,1.19)	<0.001	1.14 (1.08,1.21)	<0.001
		T1	Ref.	–	Ref.	–	Ref.	–
		T2	1.26 (1.13,1.40)	<0.001	1.25 (1.13,1.39)	<0.001	1.16 (1.04,1.30)	0.011
		T3	1.43 (1.29,1.58)	<0.001	1.45 (1.30,1.61)	<0.001	1.40 (1.24,1.58)	<0.001
	HRR and CAR	high HRR and low CAR	Ref.	–	Ref.	–	Ref.	–
		low HRR and low CAR	1.15 (0.97,1.35)	0.11	1.16 (0.98,1.37)	0.086	1.40 (1.17,1.68)	<0.001
		high HRR and high CAR	1.31 (1.18,1.46)	<0.001	1.32 (1.19,1.47)	<0.001	1.28 (1.14,1.44)	<0.001
		low HRR and high CAR	1.51 (1.34,1.70)	<0.001	1.54 (1.36,1.73)	<0.001	1.82 (1.56,2.11)	<0.001
MIMIC-III	HRR	continuous	0.52 (0.38,0.72)	<0.001	0.51 (0.37,0.71)	<0.001	0.14 (0.08,0.24)	<0.001
		T1	Ref.	–	Ref.	–	Ref.	–
		T2	0.78 (0.69,0.89)	<0.001	0.78 (0.68,0.88)	<0.001	0.65 (0.56,0.75)	<0.001
		T3	0.73 (0.64,0.83)	<0.001	0.73 (0.64,0.83)	<0.001	0.50 (0.42,0.61)	<0.001
	CAR	continuous	1.14 (1.05,1.23)	0.002	1.15 (1.06,1.24)	0.001	1.10 (1.00,1.20)	0.04
		T1	Ref.	–	Ref.	–	Ref.	–
		T2	1.22 (1.07,1.40)	0.004	1.23 (1.07,1.41)	0.004	1.15 (0.99,1.33)	0.059
		T3	1.43 (1.25,1.63)	<0.001	1.45 (1.27,1.66)	<0.001	1.27 (1.09,1.47)	0.002
	HRR and CAR	high HRR and low CAR	Ref.	–	Ref.	–	Ref.	–
		low HRR and low CAR	1.43 (1.13,1.82)	0.003	1.45 (1.14,1.83)	0.002	1.67 (1.29,2.17)	<0.001
		high HRR and high CAR	1.34 (1.19,1.51)	<0.001	1.36 (1.20,1.53)	<0.001	1.22 (1.07,1.39)	0.003
		low HRR and high CAR	1.50 (1.23,1.82)	<0.001	1.52 (1.24,1.85)	<0.001	1.47 (1.17,1.84)	0.001
eICU-CRD	HRR	continuous	0.40 (0.33,0.50)	<0.001	0.45 (0.36,0.55)	<0.001	1.33 (0.93,1.90)	0.114
		T1	Ref.	–	Ref.	–	Ref.	–
		T2	0.78 (0.71,0.85)	<0.001	0.78 (0.71,0.85)	<0.001	1.04 (0.94,1.15)	0.471
		T3	0.70 (0.63,0.77)	<0.001	0.74 (0.67,0.82)	<0.001	1.12 (0.97,1.30)	0.114
	CAR	continuous	1.08 (1.06,1.10)	<0.001	1.09 (1.07,1.10)	<0.001	1.10 (1.07,1.13)	<0.001
		T1	Ref.	–	Ref.	–	Ref.	–
		T2	1.71 (1.51,1.94)	<0.001	1.70 (1.50,1.94)	<0.001	1.59 (1.39,1.81)	<0.001
		T3	3.19 (2.84,3.57)	<0.001	3.27 (2.91,3.67)	<0.001	2.51 (2.20,2.85)	<0.001
	HRR and CAR	high HRR and low CAR	Ref.	–	Ref.	–	Ref.	–
		low HRR and low CAR	1.56 (1.20,2.04)	0.001	1.52 (1.16,1.98)	0.002	1.28 (0.97,1.68)	0.077
		high HRR and high CAR	2.61 (2.35,2.91)	<0.001	2.61 (2.34,2.92)	<0.001	2.18 (1.94,2.45)	<0.001
		low HRR and high CAR	3.62 (3.17,4.14)	<0.001	3.60 (3.15,4.12)	<0.001	2.34 (2.00,2.73)	<0.001

Model 1: Adjusted only for the main exposure variable (HRR, CAR, or joint group).

Model 2: Model 1 plus age, gender, and weight.

Model 3: Model 2 plus comorbidities (AKI, CA, CB, CKD, HEP, MI, PNA, T2DM), laboratory parameters (ALT, AST, total bilirubin, chloride, GLU, hematocrit, platelet count, RBC, WBC, sodium), GCS, and treatments (glucocorticoid use, neuromuscular blocker use, sedative or analgesic use, ventilation).

Abbreviations: HRR, hemoglobin-to-red blood cell distribution width ratio; CAR, creatinine-to-albumin ratio; HR, hazard ratio; CI, confidence interval; Ref, reference; AKI, acute kidney injury; CA, cancer; CB, chronic bronchitis; CKD, chronic kidney disease; HEP, hepatic disease; MI, myocardial infarction; PNA, pneumonia; T2DM, type 2 diabetes mellitus; GCS, Glasgow Coma Scale; RBC, red blood cell count; WBC, white blood cell count; ALT, alanine aminotransferase; AST, aspartate aminotransferase; GLU, glucose.

The predictive utility of HRR and CAR extended to longer-term outcomes across all cohorts. For 90-day mortality, higher HRR was consistently associated with risk reduction, whereas higher CAR consistently increased mortality risk (Table S5). Similar trends were observed for 180-day mortality (Table S6) and 1-year mortality (Table S7), with CAR showing particularly strong predictive value in the eICU-CRD cohort. These results underscore the temporal and cohort-specific variations in the prognostic performance of HRR and CAR.

### The association of the combined analysis of HRR and CAR with different clinical outcomes

The combined assessment of HRR and CAR demonstrated superior prognostic value compared to either indicator alone across all cohorts. In MIMIC-IV, patients with low HRR and high CAR had an 82% increased risk of 28-day mortality (HR = 1.82, 95% CI: 1.56–2.11; Table 2). This high-risk profile consistently predicted worse outcomes at 90-day, 180-day, and 1-year follow-ups, with risk increases ranging from 59% to 76% in MIMIC-IV and MIMIC-III cohorts (Tables S5–S7).

Notably, the eICU-CRD cohort showed even more pronounced results, where the low HRR/high CAR combination was associated with a 3.6-fold increased mortality risk across all time points (28-day to 1-year). These findings confirm that the HRR-CAR combination effectively identifies high-risk ICU patients requiring intensified intervention, with results remaining robust in sensitivity analyses (Table S8).

### Non-linear relationships between HRR, CAR, and various clinical outcomes

**Figure 2:**
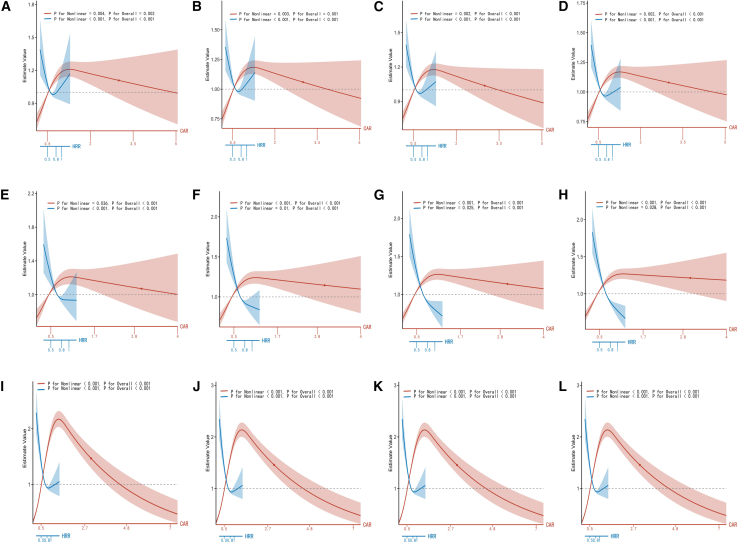
Restricted cubic spline curves for 28-day, 90-day, 180-day, and 1-year all-cause mortality stratified by HRR and CAR in MIMIC-IV, MIMIC-III, and eICU-CRD cohorts Notes: (A–D) MIMIC-IV cohort shows the associations of HRR and CAR with 28-day, 90-day, 180-day, and 1-year all-cause mortality; (E–H) MIMIC-III cohort; (I–L) eICU-CRD cohort. Abbreviations: HRR, hemoglobin-to-red cell distribution width ratio; CAR, creatinine-to-albumin ratio; MIMIC-IV/MIMIC-III, Medical Information Mart for Intensive Care IV/III; eICU-CRD, eICU Collaborative Research Database.

This study investigated the threshold effects of HRR and CAR on all-cause mortality across three independent cohorts (MIMIC-IV, MIMIC-III, eICU-CRD). Restricted cubic spline analysis confirmed non-linear relationships, with HRR exhibiting a U-shaped and CAR an inverted U-shaped association with mortality outcomes (Figure 2).

HRR demonstrated a protective effect below the threshold of 0.962, significantly reducing mortality risk in all cohorts at 28- and 90-day intervals. Conversely, values above this threshold were associated with significantly increased risk in MIMIC-IV (HR = 7.721, *p* = 0.002), though this pattern was less consistent in other cohorts for long-term outcomes.

CAR showed a distinct pattern, with values below 0.778 associated with significantly increased mortality risk across all time points. This was particularly evident in eICU-CRD (HR = 6.281, *p* < 0.001). Above this threshold, CAR did not show significant risk alteration (Table 3). These findings confirm the non-linear, threshold-dependent nature of both biomarkers in mortality risk stratification.

**Table 3:** Threshold effects of HRR and CAR on all-cause mortality at 28, 90, 180 days, and 1 year across three cohorts MIMIC-IV, MIMIC-III, and eICU-CRD

Time	Variable	Row Name	MIMIC-IV				MIMIC-III				eICU-CRD			
			HR	CI 2.5	CI 97.5	*p* value	HR	CI 2.5	CI 97.5	*p* value	HR	CI 2.5	CI 97.5	*p* value
28days	HRR	model 1 line effect	0.778	0.618	0.980	0.033	0.521	0.378	0.719	<0.001	0.402	0.326	0.495	<0.001
		model 2 threshold (W)	0.962	–	–	–	0.862	–	–	–	0.797	–	–	–
		model 2 <W effect	0.645	0.499	0.834	0.001	0.357	0.236	0.539	<0.001	0.203	0.147	0.280	<0.001
		model 2 >W effect	7.721	2.069	28.811	0.002	2.621	0.857	8.010	0.091	1.480	0.898	2.438	0.124
		log likelihood ratio test	–	–	–	0.002	–	–	–	0.005	–	–	–	<0.001
	CAR	model 1 line effect	1.114	1.058	1.173	<0.001	1.135	1.046	1.231	0.002	1.080	1.064	1.096	<0.001
		model 2 threshold (W)	0.778	–	–	0.778	0.947	–	–	0.947	1.018	–	–	1.018
		model 2 <W effect	2.094	1.676	2.615	<0.001	1.853	1.472	2.331	<0.001	6.281	5.387	7.323	<0.001
		model 2 >W effect	0.968	0.895	1.048	0.424	0.883	0.759	1.028	0.108	0.801	0.748	0.857	<0.001
		log likelihood ratio test	–	–	–	<0.001	–	–	–	<0.001	–	–	–	<0.001
90days	HRR	model 1 line effect	0.786	0.645	0.957	0.016	0.418	0.320	0.546	<0.001	0.391	0.318	0.481	<0.001
		model 2 threshold (W)	0.950	–	–	–	0.832	–	–	–	0.791	–	–	–
		model 2 <W effect	0.664	0.532	0.828	<0.001	0.292	0.204	0.419	<0.001	0.191	0.139	0.264	<0.001
		model 2 >W effect	5.427	1.769	16.653	0.003	1.414	0.607	3.293	0.422	1.460	0.896	2.380	0.129
		log likelihood ratio test	–	–	–	0.001	–	–	–	0.004	–	–	–	<0.001
	CAR	model 1 line effect	1.096	1.048	1.147	<0.001	1.166	1.091	1.246	<0.001	1.068	1.052	1.084	<0.001
		model 2 threshold (W)	0.711	–	–	–	0.947	–	–	–	1.050	–	–	–
		model 2 <W effect	2.165	1.745	2.686	<0.001	1.913	1.583	2.312	<0.001	5.656	4.891	6.540	<0.001
		model2 >W effect	0.965	0.903	1.031	0.292	0.912	0.806	1.030	0.138	0.793	0.740	0.849	<0.001
		log likelihood ratio test	–	–	–	<0.001	–	–	–	<0.001	–	–	–	<0.001
180days	HRR	model 1 line effect	0.719	0.597	0.867	0.001	0.343	0.268	0.438	<0.001	0.391	0.318	0.481	<0.001
		model 2 threshold (W)	0.951	–	–	–	0.841	–	–	–	0.791	–	–	–
		model 2 <W effect	0.588	0.478	0.725	<0.001	0.257	0.186	0.356	<0.001	0.191	0.139	0.265	<0.001
		model 2 >W effect	7.846	2.725	22.595	<0.001	1.031	0.449	2.366	0.943	1.460	0.896	2.380	0.129
		log likelihood ratio test	–	–	–	<0.001	–	–	–	0.009	–	–	–	<0.001
	CAR	model 1 line effect	1.091	1.045	1.139	<0.001	1.177	1.108	1.250	<0.001	1.068	1.052	1.084	<0.001
		model 2 threshold (W)	0.760	–	–	–	0.912	–	–	–	1.024	–	–	–
		model 2 <W effect	2.007	1.669	2.413	<0.001	2.099	1.751	2.515	<0.001	5.969	5.135	6.938	<0.001
		model 2 >W effect	0.950	0.889	1.015	0.127	0.903	0.808	1.008	0.069	0.803	0.750	0.859	<0.001
		log likelihood ratio test	–	–	–	<0.001	–	–	–	<0.001	–	–	–	<0.001
1year	HRR	model1 line effect	0.700	0.587	0.834	<0.001	0.312	0.248	0.393	<0.001	0.391	0.318	0.481	<0.001
		model 2 threshold (W)	0.962	–	–	–	0.863	–	–	–	0.791	–	–	–
		model 2 <W effect	0.597	0.492	0.725	<0.001	0.248	0.185	0.332	<0.001	0.191	0.139	0.265	<0.001
		model 2 >W effect	6.493	2.144	19.667	0.001	0.926	0.391	2.193	0.861	1.460	0.896	2.380	0.129
		log likelihood ratio test	–	–	–	<0.001	–	–	–	0.013	–	–	–	<0.001
	CAR	model 1 line effect	1.098	1.055	1.144	<0.001	1.195	1.130	1.264	<0.001	1.068	1.052	1.084	<0.001
		model 2 threshold (W)	0.818	–	–	–	0.893	–	–	–	1.024	–	–	–
		model 2 <W effect	1.774	1.516	2.075	<0.001	2.096	1.762	2.493	<0.001	5.969	5.135	6.938	<0.001
		model 2 >W effect	0.966	0.906	1.029	0.278	0.938	0.848	1.037	0.210	0.803	0.750	0.859	<0.001
		log likelihood ratio test	–	–	–	<0.001	–	–	–	<0.001	–	–	–	<0.001

Notes: Model 1: linear Cox model. Model 2: two-piecewise Cox model with a threshold (W). Log-likelihood ratio test compares Model 2 vs. Model 1; a significant *p* value indicates a nonlinear relationship with a threshold effect.

Abbreviations: HRR, hemoglobin-to-RDW ratio; CAR, creatinine-to-albumin ratio; HR, hazard ratio; CI, confidence interval; W, threshold value.

### The nonlinear relationship between HRR and CAR combined analysis and different clinical outcomes

Sensitivity analysis using median truncation confirmed the consistent association between combined HRR-CAR groups and mortality across multiple time points. In both MIMIC-IV and MIMIC-III cohorts, the low HRR and high CAR group demonstrated the highest mortality risk (MIMIC-IV: aHR 1.63–1.67; MIMIC-III: aHR 1.73–1.95, all *p* < 0.001), followed by the high HRR and high CAR group.

Notably, the eICU-CRD cohort exhibited substantially elevated risk estimates, with both high HRR and high CAR (aHR 2.38–2.40) and low HRR and high CAR groups (aHR 2.01–2.06) showing significantly higher mortality risks compared to other cohorts. While adjustments for demographic and clinical variables attenuated some associations, the overall risk stratification pattern remained robust (Table S8).

These findings underscore the clinical value of combining HRR and CAR for mortality risk assessment, with the low HRR and high CAR profile consistently identifying the highest-risk patients across diverse ICU populations.

### Survival analysis of HRR and CAR in relation to different clinical outcomes

**Figure 3:**
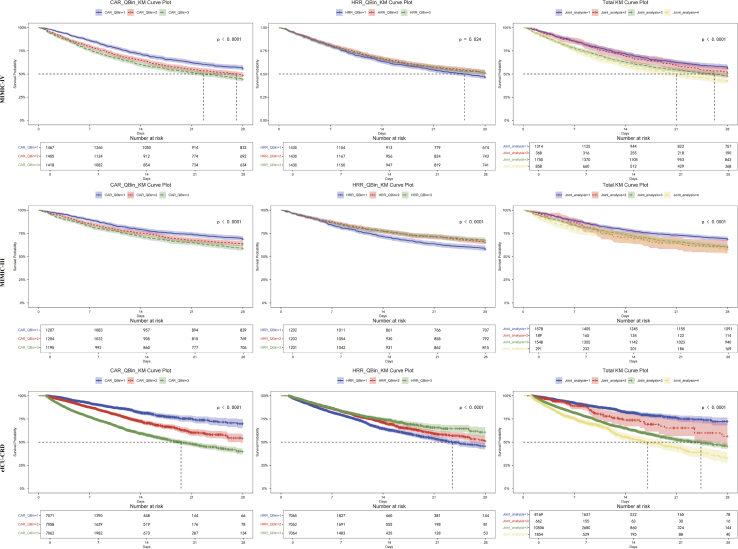
Kaplan-Meier curves for 28-day all-cause mortality stratified by CAR, HRR, and their combined effects in MIMIC-IV, MIMIC-III, and eICU-CRD Cohorts Notes: Panels show Kaplan–Meier curves for 28-day all-cause mortality in critical heart failure across three cohorts; rows (top→bottom) are MIMIC-IV, MIMIC-III, and eICU-CRD, and columns (left→right) are CAR, HRR, and their joint analysis. In the joint analysis, groups 1–4 denote, respectively, high HRR & low CAR, low HRR & low CAR, high HRR & high CAR, and low HRR & high CAR. Log rank *p*-values indicate between-group differences, and the numbers at risk at each time point are shown beneath each panel. Abbreviations: CAR, creatinine-to-albumin ratio; HRR, hemoglobin-to-red cell distribution width ratio; KM, Kaplan-Meier; ICU, intensive care unit; MIMIC-IV/MIMIC-III, Medical Information Mart for Intensive Care IV/III; eICU-CRD, eICU Collaborative Research Database.

Kaplan-Meier survival analyses based on tertiles of CAR and HRR revealed significant graded differences in 28-day cumulative survival (Figure 3). Individual analysis showed that the low CAR group had significantly higher survival rates than the medium and high CAR groups, while the low HRR group consistently demonstrated the lowest survival probability at all time points, with statistically significant differences between groups (log rank *p* < 0.001).

The combined analysis further enhanced risk stratification: The low HRR and high CAR group showed the highest mortality rate, while the high HRR and low CAR group had the most favorable survival outcome, with the other two groups exhibiting intermediate risk. The log rank test confirmed highly significant differences among the four groups (*p* < 0.001). These findings indicate that the combined assessment of CAR and HRR more accurately identifies the high-risk population for 28-day mortality (Figure 3), with the low HRR and high CAR profile representing the strongest adverse prognostic signal. This pattern remained consistent across 90-day, 180-day, and 1-year mortality outcomes (Figures S1–S3).

In the HOSP-CCU validation cohort, the combined CAR-HRR index demonstrated remarkable reliability (Figure S4). While HRR alone showed significant predictive value mainly at the 1-year time point and CAR became significant only after 90 days, their combination produced statistically significant stratification effects at all time points (28 days, 90 days, 180 days, and 1 year; all log rank *p* < 0.05). Throughout the follow-up period, the low HRR and high CAR group consistently identified patients with the highest mortality risk, confirming the unique value of the combined index for comprehensive risk assessment. This conclusion remained robust in sensitivity analysis (Figures S5 and S6).

### Predictive performance of different indicators based on AUC analysis

**Figure 4:**
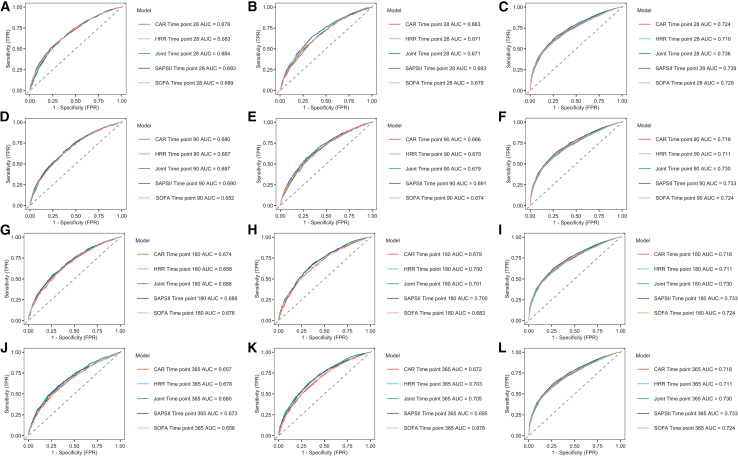
ROAUC curves for CAR, HRR, and their combination in predicting ICU mortality using Cox regression models across MIMIC-IV, MIMIC-III, and eICU-CRD cohorts Notes: The first column represents the MIMIC-IV database, the second column represents the MIMIC-III database, and the third column represents the eICU-CRD database. Each column corresponds to four time points: the first row represents the 28-day mortality rate, the second row represents the 90-day mortality rate, the third row represents the 180-day mortality rate, and the fourth row represents the 1-year mortality rate. Abbreviations: HRR: hemoglobin-to-red cell distribution width ratio; CAR: creatinine-to-albumin ratio; AUC: area under the curve; ICU: intensive care unit; MIMIC-IV/MIMIC-III: Medical Information Mart for Intensive Care IV/III; eICU-CRD: eICU Collaborative Research Database; SOFA: Sequential Organ Failure Assessment; SAPSII: Simplified Acute Physiology Score II; Joint: A four-level variable derived from HRR and CAR levels.

The HRR and CAR combination consistently yielded higher AUC values for predicting 28-day, 90-day, 180-day, and 1-year mortality than either biomarker alone across all four cohorts (Figure 4; Figure S7). To further evaluate the predictive advantage of the combined model over conventional critical illness scoring systems, we performed DeLong’s tests (Table S9) in the three large-scale ICU databases, comparing the AUCs of the combined model with those of the SOFA and SAPS II scores for all-cause mortality at multiple time points (Figure 4).

Overall, the HRR and CAR model demonstrated robust and superior discriminative ability for both short-term and long-term mortality risk prediction. In the MIMIC-IV and MIMIC-III cohorts, the predictive performance of the combined model for 28-day mortality was comparable to that of the SOFA and SAPS II scores, suggesting that it can provide early risk stratification such as traditional scoring systems shortly after ICU admission. However, the advantage of the combined model became more pronounced for long-term outcomes: In both MIMIC cohorts, its AUCs for 180-day and 1-year mortality were significantly higher than those of the SOFA score (all *p* < 0.05), indicating greater sensitivity in capturing the long-term mortality risk.

Notably, in the eICU-CRD cohort, the combined model achieved the highest AUCs across all time points (0.730–0.736). DeLong’s test confirmed that it significantly outperformed the SOFA score at all time points (all *p* < 0.01), while demonstrating non-inferior performance compared to the SAPS II score (all *p* > 0.05). These findings further validate the stability and generalizability of the combined model across diverse ICU settings and patient populations.

### Subgroup analysis

**Figure 5:**
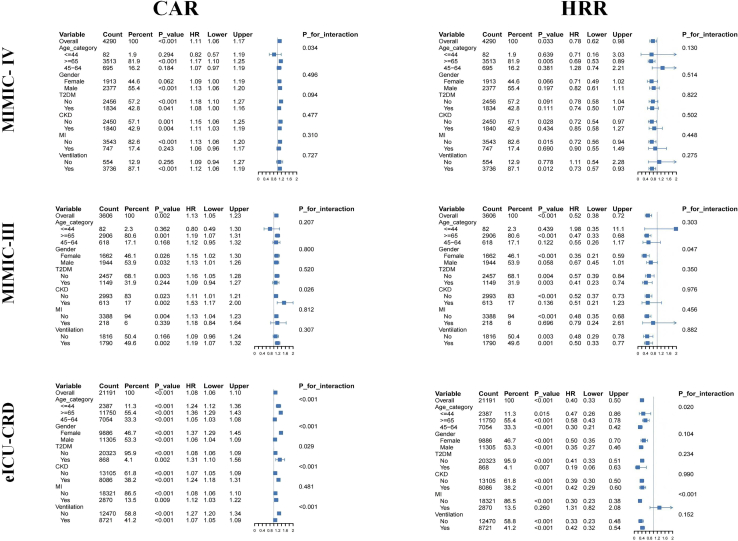
Subgroup analysis of 28-day all-cause mortality stratified by CAR and HRR in MIMIC-IV, MIMIC-III, and eICU-CRD cohorts Notes: The figure presents subgroup analyses for 28-day all-cause mortality according to CAR (left panels) and HRR (right panels) in the MIMIC-IV, MIMIC-III, and eICU-CRD cohorts. Hazard ratios (HRs) and 95% confidence intervals (CIs) were estimated using Cox proportional hazards models. *p* values for interaction are shown for each subgroup variable. Abbreviations: HR, hazard ratio; CI, confidence interval; HRR, hemoglobin-to-red cell distribution width ratio; CAR, creatinine-to-albumin ratio; T2DM, type 2 diabetes mellitus; CKD, chronic kidney disease; MI, myocardial infarction; MIMIC-IV/MIMIC-III, Medical Information Mart for Intensive Care IV/III; eICU-CRD, eICU Collaborative Research Database.

Subgroup analyses across three independent cohorts consistently demonstrated CAR as a significant risk factor and HRR as a protective factor for 28-day mortality, though effect sizes varied by population (Figure 5).

In MIMIC-IV, each 1-unit increase in CAR elevated mortality risk by 11% (HR = 1.11), with stronger effects in males and mechanically ventilated patients. MIMIC-III results showed a distinct pattern: While CAR’s overall effect was modest (HR = 1.13), HRR demonstrated strong protection (HR = 0.52), particularly in mechanically ventilated patients and females. The eICU-CRD cohort, with the largest sample size, confirmed both CAR (HR = 1.08) and HRR (HR = 0.40) as robust predictors.

For longer-term outcomes (90-day to 1-year), both biomarkers maintained significant associations with mortality, though with substantial heterogeneity across clinical subgroups. Age consistently served as a key effect modifier, with enhanced predictive performance observed in patients ≥65 years. Mechanical ventilation status and specific comorbidities (CKD, diabetes) also significantly influenced biomarker performance (Figures S8–S10).

## Discussion

The study based on three large databases (MIMIC-III, MIMIC-IV, eICU-CRD), and the results were validated by the HOSP-CCU cohort, confirms that HRR and CAR have unique prognostic judgment capabilities for critical HF populations. Among them, high HRR is an independent protective factor, while CAR is a risk factor. Patients with low HRR and high CAR have the highest 28-day mortality risk, making them an ideal high-risk screening population. Moreover, HRR and CAR have cumulative prognostic stratification value. We also confirmed the robustness of the results through sensitivity analysis and subgroup analysis, suggesting that the combined application of these two indicators can better identify high-risk patients, provide immediate death risk warnings for critical patients with HF, and directly guide clinical medical staff to strengthen monitoring and implement anti-inflammatory-nutrition combined interventions early.

We Further emphasize the relationship between inflammatory markers and the prognosis of critical HF. Similarly, other studies have also confirmed the potential association between inflammation and HF. Inflammation and HF are a bidirectional process.^22^ Acute HF episodes are typically accompanied by the release of substantial cytokines and the activation of the immune system, which may exacerbate myocardial injury and promote ventricular remodeling. Elevated CAR reflects renal impairment and hypoalbuminemia, both of which can trigger and amplify inflammatory responses, oxidative stress, and apoptosis, thereby accelerating HF progression.^20^^,^^23^ During the inflammatory process, the immune system is often overactivated, and these immunological alterations, along with excessive cytokine release and play a pivotal role in the clinical manifestation of HF.^24^^,^^25^ Hemoglobin (Hb) reflects malnutrition and systemic inflammation, while red cell distribution width (RDW) has been identified as a predictor of various cardiovascular diseases and is increasingly recognized as a significant prognostic marker in HF. The combination of Hb and RDW may offer superior predictive value compared to either marker alone.^26^ Anemia of inflammation, driven by inflammatory cytokines such as tumor necrosis factor-alpha and interleukins, is another form of anemia commonly observed in HF and is closely associated with HRR.^27^ These cytokines are also linked to poor clinical outcomes.^28^^,^^29^ Therefore, CAR and HRR may not only serve as indicators of renal function and nutritional status but also provide deeper insights into the underlying pathophysiological processes of HF, offering a more comprehensive assessment of patient condition.

In populations with HF, Hb is often below normal levels and has been proven to be an independent predictor of HF.^30^^,^^31^ Additionally, changes in RDW are closely related to the body’s inflammatory response and play a certain prognostic role for various organs such as the heart, liver, and brain, especially in terms of cardiovascular health.^32^^,^^33^^,^^34^ By coupling Cr and ALB, a dynamic integrated indicator that surpasses the information content of a single indicator is constructed. This indicator has a more significant impact on postoperative risk assessment.^35^^,^^36^ The study indicates that using Cr or ALB alone is less effective than CAR in predicting mortality. The interplay among multiple organ systems in patients with HF is highly complex. CAR may reflect early-stage cardio-renal dysfunction, as well as persistent inflammation and nutritional depletion, which are factors that independently drive adverse outcomes even in the absence of clinically diagnosed renal conditions such as acute kidney injury or chronic kidney disease.^29^^,^^37^ Notably, these associations remained significant in our study after adjusting for relevant covariates. HRR and CAR are considered powerful indicators in judging disease prognosis, including for patients with cardiovascular adverse conditions,^21^^,^^38^^,^^39^^,^^40^which is consistent with the results of this study. While previous studies have utilized HRR and the CAR to predict prognosis in patients with HF, these investigations were confined to single-center databases and focused solely on HF-related mortality.^41^^,^^42^ Consequently, their findings lack external validation, limiting the generalizability of the results.

This research demonstrates the unique clinical value of two composite biomarkers in assessing the prognosis of ICU patients with HF. Compared with recent studies focusing on acute cardiovascular emergencies, which, despite affirming the predictive role of inflammatory parameters such as the systemic immune-inflammation index (SII), systemic inflammation response index (SIRI), neutrophil-to-lymphocyte ratio (NLR), platelet-to-lymphocyte ratio (PLR), and multiple inflammation indices (MII-1, MII-2, MII-3) in early emergency triage for predicting discharge and mortality outcomes, their perspective remained largely confined to single inflammatory pathways.^43^^,^^44^ Furthermore, although Chen et al. explored the use of HRR in evaluating mortality and readmission rates in patients with HF, their study population predominantly consisted of relatively stable patients in general wards or outpatient follow-up settings, lacking the context of extreme catabolism and hemodynamic fluctuations characteristic of the ICU environment.^45^^,^^46^ In contrast, the present study extends the observation window from initial emergency triage to the more pathophysiological complex ICU phase. The selected HRR and CAR biomarkers encompass not only inflammatory information but also integrate nutritional status—a factor that, for critically ill patients with HF experiencing stress, induced decompensation with exceptionally high metabolic demands, offers greater pathophysiological explanatory power than inflammatory cell counts alone.

Subgroup analyses showed that, overall, HRR was more strongly associated in older women, whereas CAR was more prominently associated in older men. Interestingly, the present study combined four time points (28 days, 90 days, 180 days, and 1 year), and all four nodes consistently showed that high HRR was a protective factor independent of traditional variables in critical patients with HF, and the effect was strongest in the early stages and stabilized in the later stages, while high CAR was a persistent risk factor, with an increasing trend of adverse effects with follow-up time, which was a complementary relationship. In the elderly population, due to various factors such as underlying diseases and metabolism, inflammatory mediators can lead to erythrocyte destruction and inhibit erythropoiesis, thus affecting RDW and exacerbating oxygen transport abnormalities; the protective effect of HRR may be reduced, and the degree of risk of HF is increased.^47^ At the same time, high Cr is positively correlated with the risk of heart,^40^ and serum albumin, an objective indicator of nutritional status and clinical inflammation, is down-regulated during inflammation. downregulated in inflammation.

Integrating longitudinal monitoring, HF phenotype heterogeneity, and the expansion of existing prognostic frameworks may further enhance the clinical translational value of HRR and CAR. In this study, HRR and CAR demonstrated prognostic performance comparable to established ICU severity scores such as SOFA and SAPS II, with the added advantage of being derived from routinely available laboratory parameters. First, although the present analysis was based on single measurements obtained at ICU admission, which may not fully capture the dynamic fluctuations in inflammatory status, renal function, and nutritional reserve throughout the course of HF, future studies incorporating longitudinal trajectories of these biomarkers through serial measurements or continuous assessment via wearable devices could substantially improve risk prediction in critically ill patients.^48^^,^^49^ Second, traditional prognostic assessment in HF has established a framework centered on BNP or NT-proBNP, integrated with clinical risk scores and imaging parameters; however, existing tools exhibit blind spots in capturing multidimensional information related to inflammation,^50^ nutrition, and renal function, and by introducing HRR and CAR as complementary biomarkers, this study supports a multi-marker integration strategy that augments rather than replaces existing frameworks, thereby enabling more comprehensive risk stratification.^51^ Third, both HRR and CAR are derived from routine laboratory tests obtained within 24 h of ICU admission, incurring no additional cost and offering inherent advantages for clinical integration as early warning indicators available at the time of ICU entry, helping identify patients who may benefit from closer monitoring, nutritional optimization, or renal protective strategies, and this low-cost, high-efficiency screening model holds strong translational potential particularly in acute critical care settings where resources are limited and rapid decision-making is essential, thereby supporting precision management of patients with HF.^52^ Finally, it is important to emphasize that HF phenotype heterogeneity may influence biomarker performance, as studies have shown that abnormal calcium homeostasis in cardiomyocytes is a hallmark pathogenic mechanism in HF and therapeutic interventions targeting this intracellular process, such as beta-blockers, have proven beneficial in HF with reduced ejection fraction but ineffective in HF with preserved ejection fraction.^53^ Therefore, future research should systematically investigate the differential mechanisms and predictive value of HRR and CAR across distinct HF subtypes.

There are some insights into inflammatory biomarkers provides valuable guidance for predicting poor prognosis in critical patients with HF in this study. First, this study integrates three major databases, MIMIC-III, MIMIC-IV, and eICU, and it was also verified using the hospital database, which is validated across geographic regions and is externally generalizable. Secondly, HRR and CAR were calculated from routine blood tests within 24 h of admission, with no additional cost, and were immediately available in the clinic. Lastly, the follow-up of this study was complete, covering four nodes: 28 days, 90 days, 180 days, and 1 year, which systematically depicted the time-risk relationship.

### Limitations of the study

As an observational study, causal inference is limited; unobserved confounders may affect the relationship between HRR and CAR and HF, and different countries’ social, economic, and cultural backgrounds and healthcare systems may influence the results. Despite the integration of multinational databases in this study, regional heterogeneity in healthcare resource allocation, care accessibility, and population health behaviors may still limit the comparability of cross-national results. Third, HRR and CAR were assessed solely based on laboratory and physiological data obtained within the first 24 h of ICU admission. Although this approach facilitates early risk stratification, it fails to capture the dynamic fluctuations of these markers over the course of ICU stay.

## Resource availability

### Lead contact

Jianghua Zhou (zhoujianghua@wmu.edu.cn).

### Materials availability

This study did not generate new unique reagents or materials.

### Data and code availability

• The data from the First Affiliated Hospital of Wenzhou Medical University are not publicly available due to privacy and ethical restrictions. But they are available from the corresponding author, J.Z., upon reasonable request. The MIMIC-III, MIMIC-IV, and eICU datasets are publicly available from PhysioNet (https://physionet.org/), subject to data use agreements.
• This paper does not report original code.
• Any additional information required to reanalyze the data reported in this paper is available from the lead contact upon request

## Acknowledgments

This work was supported by the General Program of the 10.13039/501100014996Health Commission of Zhejiang Province (grant no. 2024KY1272). The authors thank the developers and maintainers of the MIMIC-III, MIMIC-IV, eICU-CRD, and Hospital cohort databases for creating and sharing these invaluable open-access critical care resources.

## Author contributions

Conceptualization and supervision: J.Z. ; methodology: J.Z., F.C., P.H., and H.D.; data curation: S.C., F.W., and Q.L. ; formal analysis: S.C. and F.W. ; investigation: S.C., F.W., B.C., and M.Z. ; validation: F.C., P.H., J.Z., and H.D. ; visualization: S.C., B.C., Q.L., and J.Y.; writing – original draft: B.C., M.Z., and J.Y.; writing – review and editing: F.C., P.H., J.Z., and H.D.; project administration: F.C., P.H., J.Z., and H.D.; software: S.C., F.W., B.C., J.Z., and H.D. Final approval of the manuscript: All authors equal contribution: S.C., F.W., and B.C. contributed equally to this work. All authors had access to the underlying data, participated in data interpretation, reviewed, and approved the final version of the manuscript, and agree to be accountable for all aspects of the work.

## Declaration of interests

The authors declare no competing interests.

## STAR★Methods

### Key resources table

**Table:** 

REAGENT or RESOURCE	SOURCE	IDENTIFIER
**Deposited data**		

Patients at the Coronary Care Unit of the First Affiliated Hospital of Wenzhou Medical University (2015–2024)	This paper	The data from the First Affiliated Hospital of Wenzhou Medical University are not publicly available due to privacy and ethical restrictions. But are available from the corresponding author J.Z. upon reasonable request.
Medical Information Mart for Intensive Care-IV	Physionet	https://physionet.org/content/mimiciv/3.1/
Medical Information Mart for Intensive Care-III	Physionet	https://physionet.org/content/mimiciii/1.4/
eICU Collaborative Research Database	Physionet	https://physionet.org/content/eicu-crd/2.0/

**Software and algorithms**		

R (version 4.4.1)	The R Foundation	https://www.r-project.org
DecisionLinnc (V1.1.6.8)	StatsApe	https://www.statsape.com/

### Experimental model and study participant details

This study leveraged data from three public critical care databases. The Medical Information Mart for Intensive Care IV (MIMIC-IV, v2.0; 2008 - 2019), MIMIC-III (v1.4; 2001 - 2012), and the eICU Collaborative Research Database (eICU-CRD, v2.0; 2014 - 2015), which constituted the primary cohorts for analysis.^22^^,^^30^^,^^40^^,^^54^ To ensure the independence of the cohort, we strictly excluded patients in MIMIC-III who overlapped with those in MIMIC-IV based on the unique identifier (subject_id). The robustness of the findings was subsequently further evaluated in an independent external validation cohort (2015-2024) from the Coronary Care Unit (CCU) of the First Affiliated Hospital of Wenzhou Medical University. This research strictly adhered to the Helsinki Declaration. All patient information in the open databases of MIMIC-IV, MIMIC-III, and eICU-CRD has been anonymized, and the use of the data has been certified by the CITI project (record ID: 64298950). Additionally, for the hospital cohort involved in this study, the relevant research protocol has been reviewed and approved by the Ethics Committee of the First Affiliated Hospital of Wenzhou Medical University (approval number: 2024KY1272).

### Method details

#### Study design and population

We included all adult patients in the intensive care units (ICUs) who had ICD-9 or ICD-10 codes related to heart failure in the MIMIC-IV, MIMIC-III and eICU-CRD databases, as well as heart failure patients from the CCU cohort of the First Affiliated Hospital of Wenzhou Medical University. Exclusion criteria were: (1) age less than 18 years; (2) multiple ICU admissions (only considering the first admission); (3) ICU stay less than 24 hours; (4) missing death records or ICU admission/discharge times, hemoglobin, red blood cell distribution width, creatinine or albumin records. After applying these criteria, the final analysis included 4,290 participants from MIMIC-IV, 3,606 participants from MIMIC-III, 21,192 participants from eICU-CRD, and 2,366 participants from the hospital cohort (Figure 1; Figure S11).

#### Data extraction

We extracted the following data: demographic characteristics (age, gender, weight), comorbidities (diabetes, hypertension, chronic kidney disease, acute kidney injury, myocardial infarction, cerebrovascular accident, pneumonia, cancer, hyperlipidemia, hepatitis, liver cirrhosis, chronic bronchitis), severity score at ICU admission (SAPSII, SOFA), laboratory test results (within 24 hours after admission to ICU, including hemoglobin, hematocrit, white blood cell count, platelet count, red blood cell count, red blood cell distribution width, albumin, liver enzymes, total bilirubin, blood urea nitrogen, creatinine, blood calcium, blood potassium, blood sodium, blood nitrogen, anion gap, blood glucose), intervention measures (mechanical ventilation) and drug treatments (insulin, B receptor blockers, neuromuscular blockers, sedative analgesics, vasopressin, glucocorticoids, angiotensin-converting enzyme inhibitors, angiotensin receptor blockers). For the repeatedly measured laboratory and vital sign data, the first measured values after admission to ICU were selected for analysis.

#### Definition

The HRR and CAR formulas are as follows: HRR = Hb/RDW,^16^^,^^21^ CAR = Cr/ALB.^25^ HRR is defined as the ratio of hemoglobin (Hb) to red cell distribution width (RDW) (HRR = Hb / RDW), and CAR is the ratio of serum creatinine (Cr) to albumin (ALB) (CAR = Cr / ALB).

#### Clinical outcomes

The primary outcome was 28-day all-cause mortality after ICU admission; secondary outcomes included 90-day, 180-day, and 1-year all-cause mortality. Given the differences in follow-up mechanisms among the various databases, the eICU cohort adopted right-censoring for patients who survived and were discharged; while the MIMIC database provided reliable long-term follow-up data by linking with the state death registration system. The outcomes for the HOSP-CCU cohort were tracked through standardized phone follow-ups managed by cardiologists and their teams, along with the hospital's electronic health record system.

### Quantification and statistical analysis

A normality test was conducted for continuous variables. Normally distributed data were presented as mean ± standard deviation, while skewed data were expressed as median (interquartile range). Categorical variables were presented as frequencies (percentages). Comparisons between groups were performed using the Kruskal-Walli’s test or chi-square test. Regarding missing data, covariates with missing values greater than 40% were excluded, and the remaining missing data were handled using multiple imputation by chained equations (MICE) (Figures S12–S14).

The Cox proportional hazards regression model was used to evaluate the associations between HRR, CAR, and clinical outcomes. Multicollinearity among adjusted variables was assessed using the Variance Inflation Factor (VIF) (Table S10). For HRR and CAR, patients were grouped into tertiles. For the combined HRR-CAR analysis, patients were stratified into high- or low-risk groups based on optimal cut-off points determined using Kaplan-Meier survival analysis and the Log-Rank Test. Due to the inherent lack of systematic post-discharge follow-up in the eICU-CRD database, patients discharged alive in this cohort were right-censored at the time of hospital discharge for all long-term mortality analyses.

The Kaplan-Meier survival curve and log-rank test were used to compare cumulative incidences across stratifications. The AUC values were derived from Cox regression models to evaluate the predictive performance of indicators across the three clinical datasets. Subgroup analyses and interaction tests were conducted based on age, gender, and comorbidities. Finally, sensitivity analyses were performed using median-based truncation models and combined-effect models to verify the robustness of our findings across different clinical outcomes. All statistical analyses were performed using R (v4.4.1) and DecisionLinnc (v1.1.6.8). Statistical significance was defined as a two-sided *p* < 0.05.

## Supplemental information

Document S1. Figures S1–S14 and Tables S5–S7, S9, and S10

Table S1. Baseline characteristics of patients in the MIMIC-IV database according to HRR and CAR tertiles Abbreviations: Q1, first quartile; Q3, third quartile; Alb, albumin; ALT, alanine aminotransferase; AST, aspartate aminotransferase; BUN, blood urea nitrogen; CAR, creatinine-to-albumin ratio; Cr, creatinine; Glu, glucose; Hb, hemoglobin; HRR, hemoglobin to red cell distribution width ratio; Los, length of stay; RBC, red blood cell count; RDW, red cell distribution width; SAPSII, Simplified Acute Physiology Score II; SOFA, Sequential Organ Failure Assessment; WBC, white blood cell count; GCS, Glasgow Coma Scale; QBin, quartile classification; ACEI, angiotensin-converting enzyme inhibitors; ARB, angiotensin receptor blockers; CB, chronic bronchitis; MI, myocardial infarction; HLD, hyperlipidemia; T2DM, type 2 diabetes mellitus; CA, cancer; CKD, chronic kidney disease; CVA, cerebrovascular accident; PNA, pneumonia; HEP, hepatitis; LC, liver cirrhosis; AKI, acute kidney injury; HTN, hypertension.

Table S2. Baseline characteristics of patients in the MIMIC-III database according to HRR and CAR tertiles Abbreviations: Q1, first quartile; Q3, third quartile; Alb, albumin; ALT, alanine aminotransferase; AST, aspartate aminotransferase; BUN, blood urea nitrogen; CAR, creatinine-to-albumin ratio; Cr, creatinine; Glu, glucose; Hb, hemoglobin; HRR, hemoglobin to red cell distribution width ratio; Los, length of stay; RBC, red blood cell count; RDW, red cell distribution width; SAPSII, Simplified Acute Physiology Score II; SOFA, Sequential Organ Failure Assessment; WBC, white blood cell count; GCS, Glasgow Coma Scale; QBin, quartile classification; ACEI, angiotensin-converting enzyme inhibitors; ARB, angiotensin receptor blockers; CB, chronic bronchitis; MI, myocardial infarction; HLD, hyperlipidemia; T2DM, type 2 diabetes mellitus; CA, cancer; CKD, chronic kidney disease; CVA, cerebrovascular accident; PNA, pneumonia; HEP, hepatitis; LC, liver cirrhosis; AKI, acute kidney injury; HTN, hypertension.

Table S3. Baseline characteristics of patients in the eICU-CRD database according to HRR and CAR Tertiles Abbreviations: Q1, first quartile; Q3, third quartile; Alb, albumin; ALT, alanine aminotransferase; AST, aspartate aminotransferase; BUN, blood urea nitrogen; CAR, creatinine-to-albumin ratio; Cr, creatinine; Glu, glucose; Hb, hemoglobin; HRR, hemoglobin to red cell distribution width ratio; Los, length of stay; RBC, red blood cell count; RDW, red cell distribution width; SAPSII, Simplified Acute Physiology Score II; SOFA, sequential organ failure assessment; WBC, white blood cell count; GCS, Glasgow Coma Scale; QBin, quartile classification; ACEI, angiotensin-converting enzyme inhibitors; ARB, angiotensin receptor blockers; CB, chronic bronchitis; MI, myocardial infarction; HLD, hyperlipidemia; T2DM, type 2 diabetes mellitus; CA, cancer; CKD, chronic kidney disease; CVA, cerebrovascular accident; PNA, pneumonia; HEP, hepatitis; LC, liver cirrhosis; AKI, acute kidney injury; HTN, hypertension.

Table S4. Baseline Characteristics of patients in the HOSP-CCU according to HRR and CAR Abbreviations: Q1, first quartile; Q3, third quartile; Alb, Albumin; ALT, Alanine Aminotransferase; AST, Aspartate Aminotransferase; CAR, Creatinine to Albumin Ratio; Cr, Creatinine; Glu, Glucose; Hb, Hemoglobin; HRR, hemoglobin to red cell distribution width ratio; Los, length of stay; RBC, red blood cell count; RDW, red cell distribution width; WBC, white blood cell count; CB, chronic bronchitis; MI, myocardial infarction; T2DM, type 2 diabetes mellitus; CA, cancer; CKD, chronic kidney disease; PNA, pneumonia; HEP, hepatitis; AKI, acute kidney injury; HTN, hypertension.

Table S8. Sensitivity analysis using median cutoff association of HRR and CAR joint groups with 28 days, 90 days, 180 days, and 1-year mortality in MIMIC-IV, MIMIC-III, and eICU-CRD Cohorts Notes: Stratification is based on median cut points—HRR and CAR are dichotomized at their median values for the joint analysis. Model 1: Adjusted only for the main exposure variable (HRR, CAR, or joint group). Model 2: Model 1 plus age, gender, and weight. Model 3: Model 2 plus comorbidities (AKI, CA, CB, CKD, HEP, MI, PNA, T2DM), laboratory parameters (ALT, AST, total bilirubin, chloride, GLU, hematocrit, platelet count, RBC, WBC, sodium), GCS, and treatments (glucocorticoid use, neuromuscular blocker use, sedative or analgesic use, ventilation). Abbreviations: HRR, hemoglobin-to-red blood cell distribution width ratio; CAR, creatinine-to-albumin ratio; HR, hazard ratio; CI, confidence interval; Ref, reference; AKI, acute kidney injury; CA, cancer; CB, chronic bronchitis; CKD, chronic kidney disease; HEP, hepatic disease; MI, myocardial infarction; PNA, pneumonia; T2DM, type 2 diabetes mellitus; GCS, Glasgow Coma Scale; RBC, red blood cell count; WBC, white blood cell count; ALT, alanine aminotransferase; AST, aspartate aminotransferase; GLU, glucose.
