# Supplementary material for: Joint analysis of hemoglobin-to-RDW and creatinine-to-albumin ratios for mortality prediction in critical heart failure
Source: iScience. 2026 Apr 17;29(5):115740. doi: 10.1016/j.isci.2026.115740 (PMC13156559; doi:10.1016/j.isci.2026.115740)
Supplement: Document S1. Figures S1–S14 and Tables S5–S7, S9, and S10 [file mmc1.pdf]

iScience, Volume *29*

Supplemental information

Joint analysis of hemoglobin-to-RDW and creatinine-to-albumin ratios for mortality prediction in critical heart failure

Shengzhang Chen, Fei Wang, Binyan Chen, Qian Lu, Miwen Zou, Jiaying Lou, Fuman Cai, Pan Huang, Jianghua Zhou, and Haodi Dang

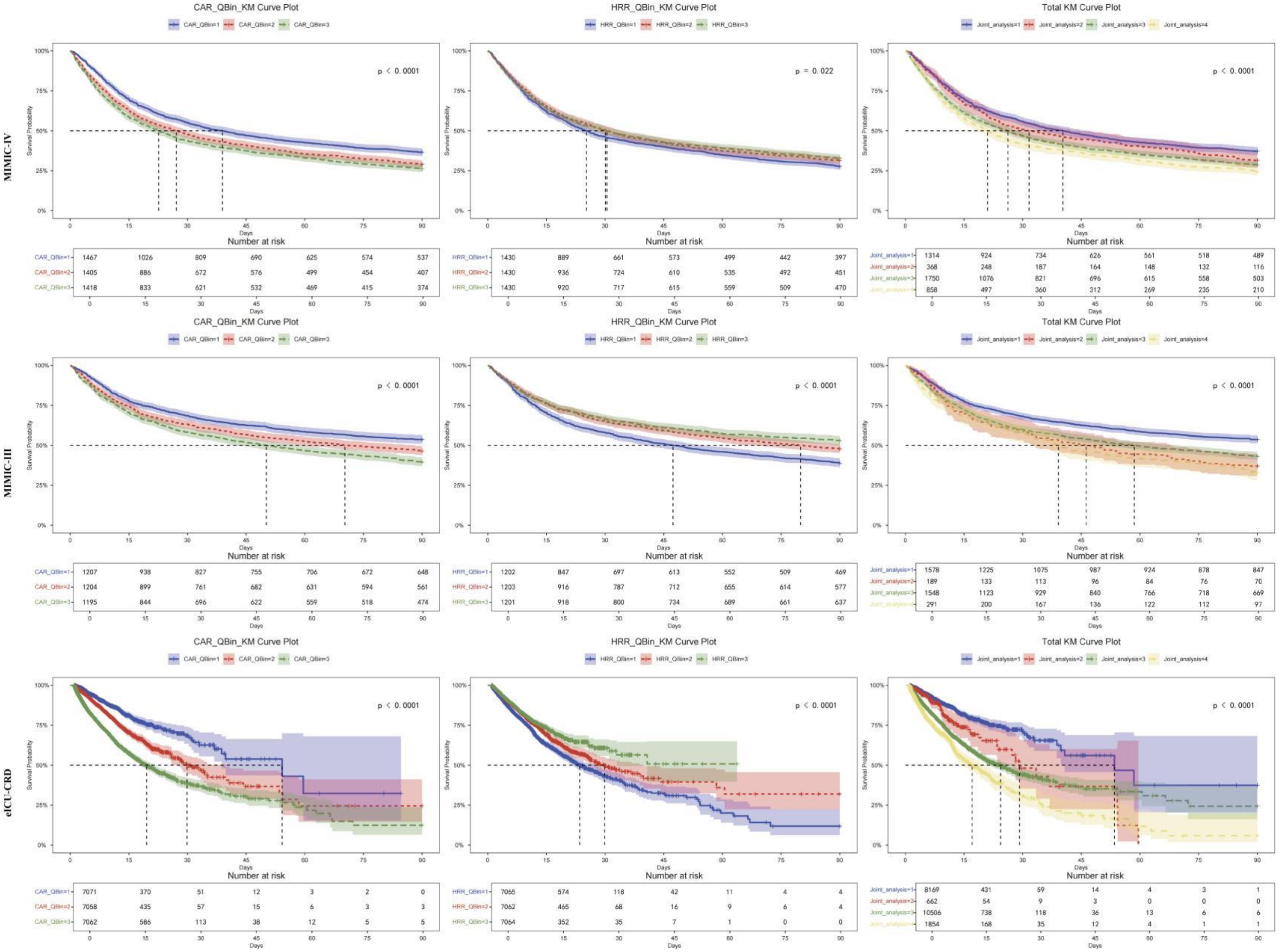
MIMIC-IV
MIMIC-III
eICU-CRD
CAR_QBin_KM Curve Plot
CAR_QBin=1 CAR_QBin=2 CAR_QBin=3
HRR_QBin_KM Curve Plot
HRR_QBin=1 HRR_QBin=2 HRR_QBin=3
Total KM Curve Plot
Joint_analysis=1 Joint_analysis=2 Joint_analysis=3 Joint_analysis=4
Survival Probability
Days
Number at risk
p < 0.0001
p = 0.022
p < 0.0001
p < 0.0001
p < 0.0001
p < 0.0001
p < 0.0001
p < 0.0001
p < 0.0001
CAR_QBin=1 1467 1026 809 690 625 574 537
CAR_QBin=2 1405 886 672 576 499 454 407
CAR_QBin=3 1418 833 621 532 469 415 374
HRR_QBin=1 1430 889 661 573 499 442 397
HRR_QBin=2 1430 936 724 610 535 492 451
HRR_QBin=3 1430 920 717 615 559 509 470
Joint_analysis=1 1314 924 734 626 561 518 489
Joint_analysis=2 368 248 187 164 148 132 116
Joint_analysis=3 1750 1076 821 696 615 558 503
Joint_analysis=4 858 497 360 312 269 235 210
CAR_QBin=1 1207 938 827 755 706 672 648
CAR_QBin=2 1204 899 761 682 631 594 561
CAR_QBin=3 1195 844 696 622 559 518 474
HRR_QBin=1 1202 847 697 613 552 509 469
HRR_QBin=2 1203 916 787 712 655 614 577
HRR_QBin=3 1201 918 800 734 689 661 637
Joint_analysis=1 1578 1225 1075 987 924 878 847
Joint_analysis=2 189 133 113 96 84 76 70
Joint_analysis=3 1548 1123 929 840 766 718 669
Joint_analysis=4 291 200 167 136 122 112 97
CAR_QBin=1 7071 370 51 12 3 2 0
CAR_QBin=2 7058 435 57 15 6 3 3
CAR_QBin=3 7062 586 113 38 12 5 5
HRR_QBin=1 7065 574 118 42 11 4 4
HRR_QBin=2 7062 465 68 16 9 6 4
HRR_QBin=3 7064 352 35 7 1 0 0
Joint_analysis=1 8169 431 59 14 4 3 1
Joint_analysis=2 662 54 9 3 0 0 0
Joint_analysis=3 10506 738 118 36 13 6 6
Joint_analysis=4 1854 168 35 12 4 1 1

Figure S1. Kaplan-Meier Curves for 90-Day All-Cause Mortality Stratified by CAR, HRR, and Their Combined Effects in MIMIC-IV, MIMIC-III, and eICU-CRD Cohorts

Notes: Panels show Kaplan–Meier curves for 90-day all-cause mortality in critical heart failure across three cohorts; rows (top→bottom) are MIMIC-IV, MIMIC-III, and eICU-CRD, and columns (left→right) are CAR, HRR, and their joint analysis. In the joint analysis, groups 1–4 denote, respectively, High HRR & Low CAR, Low HRR & Low CAR, High HRR & High CAR, and Low HRR & High CAR. Log-rank *p*-values indicate between-group differences, and the numbers at risk at each time point are shown beneath each panel.

Abbreviations: CAR, creatinine-to-albumin ratio; HRR, hemoglobin-to-red cell distribution width ratio; KM, Kaplan-Meier; ICU, intensive care unit; MIMIC-IV/MIMIC-III, Medical Information Mart for Intensive Care IV/III; eICU-CRD, eICU Collaborative Research Database.

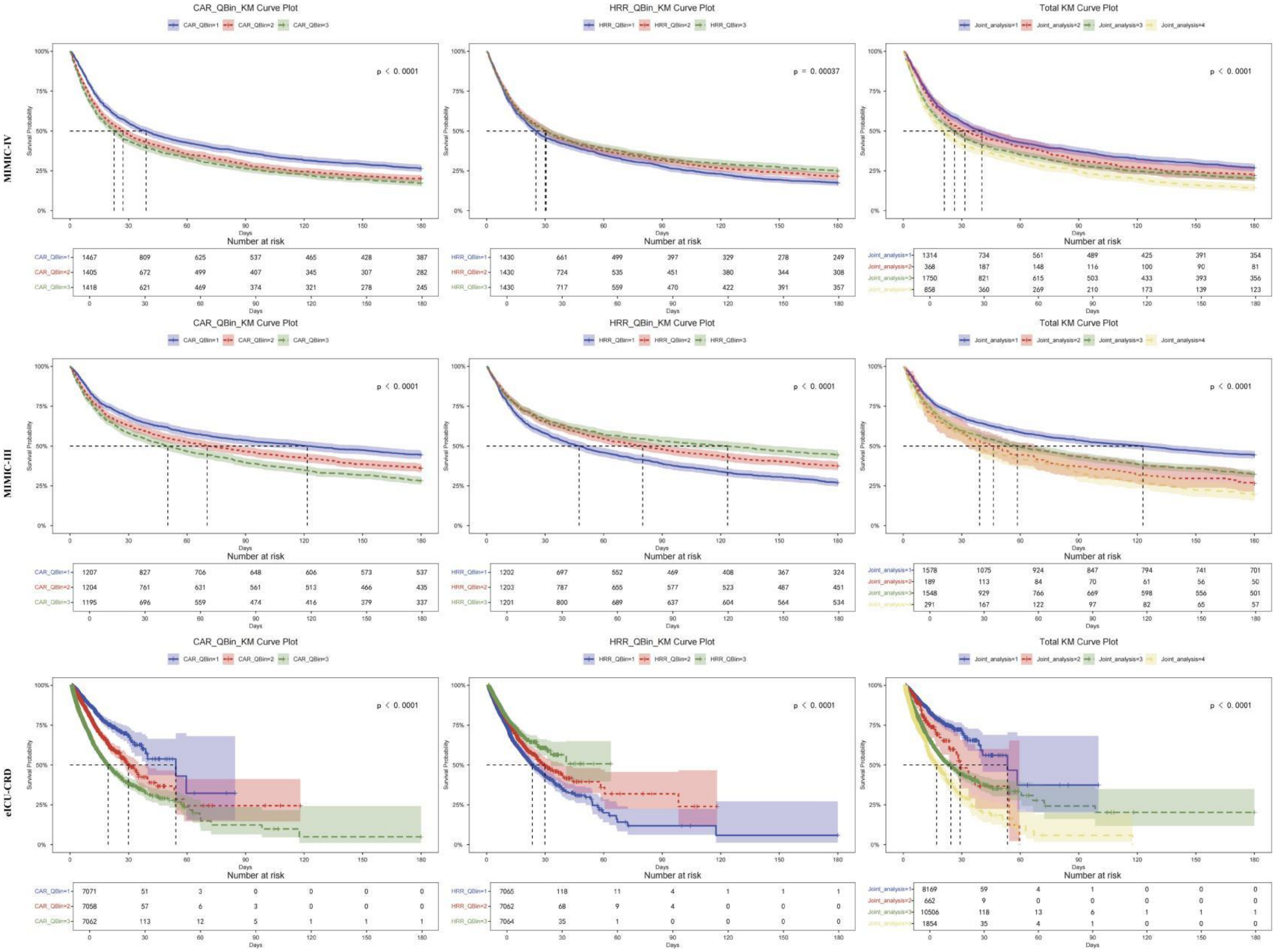
MIMIC-IV
MIMIC-III
eICU-CRD
CAR_QBin_KM Curve Plot
CAR_QBin=1 CAR_QBin=2 CAR_QBin=3
HRR_QBin_KM Curve Plot
HRR_QBin=1 HRR_QBin=2 HRR_QBin=3
Total KM Curve Plot
Joint_analysis=1 Joint_analysis=2 Joint_analysis=3 Joint_analysis=4
Survival Probability
Days
Number at risk
p < 0.0001
p = 0.00037
CAR_QBin=1 1467 809 625 537 465 428 387
CAR_QBin=2 1405 672 499 407 345 307 282
CAR_QBin=3 1418 621 469 374 321 278 245
HRR_QBin=1 1430 661 499 397 329 278 249
HRR_QBin=2 1430 724 535 451 380 344 308
HRR_QBin=3 1430 717 559 470 422 391 357
Joint_analysis=1 1314 734 561 489 425 391 354
Joint_analysis=2 368 187 148 116 100 90 81
Joint_analysis=3 1750 821 615 503 433 393 356
Joint_analysis=4 858 360 269 210 173 139 123
CAR_QBin=1 1207 827 706 648 606 573 537
CAR_QBin=2 1204 761 631 561 513 466 435
CAR_QBin=3 1195 696 559 474 416 379 337
HRR_QBin=1 1202 697 552 469 408 367 324
HRR_QBin=2 1203 787 655 577 523 487 451
HRR_QBin=3 1201 800 689 637 604 564 534
Joint_analysis=1 1578 1075 924 847 794 741 701
Joint_analysis=2 189 113 84 70 61 56 50
Joint_analysis=3 1548 929 766 669 598 556 501
Joint_analysis=4 291 167 122 97 82 65 57
CAR_QBin=1 7071 51 3 0 0 0 0
CAR_QBin=2 7058 57 6 3 0 0 0
CAR_QBin=3 7062 113 12 5 1 1 1
HRR_QBin=1 7065 118 11 4 1 1 1
HRR_QBin=2 7062 68 9 4 0 0 0
HRR_QBin=3 7064 35 1 0 0 0 0
Joint_analysis=1 8169 59 4 1 0 0 0
Joint_analysis=2 662 9 0 0 0 0 0
Joint_analysis=3 10506 118 13 6 1 1 1
Joint_analysis=4 1854 35 4 1 0 0 0

Figure S2. Kaplan-Meier Curves for 180-Day All-Cause Mortality Stratified by CAR, HRR, and Their Combined Effects in MIMIC-IV, MIMIC-III, and eICU-CRD Cohorts

Notes: Panels show Kaplan–Meier curves for 180-day all-cause mortality in critical heart failure across three cohorts; rows (top→bottom) are MIMIC-IV, MIMIC-III, and eICU-CRD, and columns (left→right) are CAR, HRR, and their joint analysis. In the joint analysis, groups 1–4 denote, respectively, High HRR & Low CAR, Low HRR & Low CAR, High HRR & High CAR, and Low HRR & High CAR. Log-rank *p*-values indicate between-group differences, and the numbers at risk at each time point are shown beneath each panel.

Abbreviations: CAR, creatinine-to-albumin ratio; HRR, hemoglobin-to-red cell distribution width ratio; KM, Kaplan-Meier; ICU, intensive care unit; MIMIC-IV/MIMIC-III, Medical Information Mart for Intensive Care IV/III; eICU-CRD, eICU Collaborative Research Database.

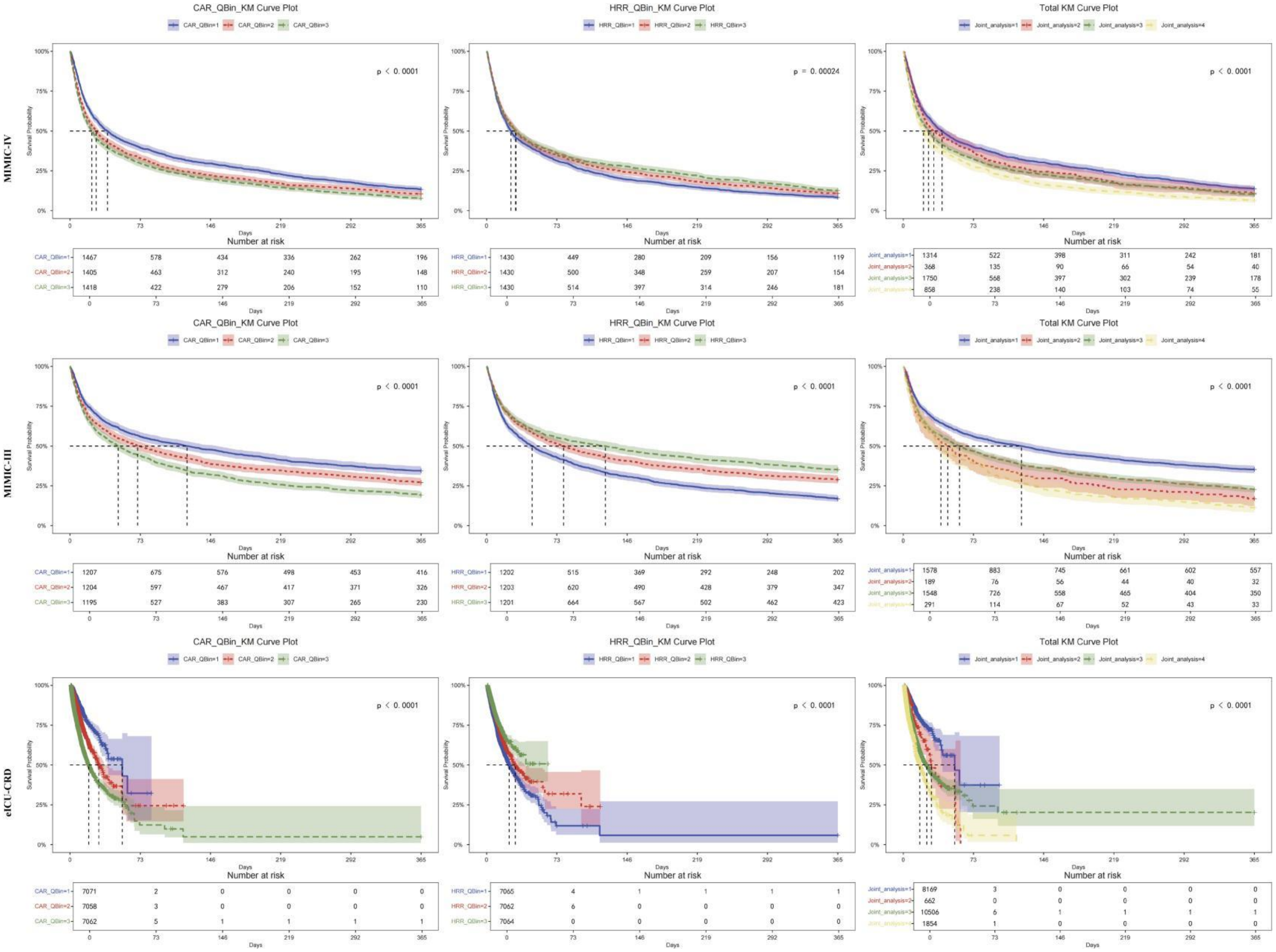
MIMIC-IV
MIMIC-III
eICU-CRD
CAR_QBin_KM Curve Plot
CAR_QBin=1 CAR_QBin=2 CAR_QBin=3
HRR_QBin_KM Curve Plot
HRR_QBin=1 HRR_QBin=2 HRR_QBin=3
Total KM Curve Plot
Joint_analysis=1 Joint_analysis=2 Joint_analysis=3 Joint_analysis=4
Survival Probability
100%
75%
50%
25%
0%
Days
0 73 146 219 292 365
p < 0.0001
p = 0.00024
Number at risk
CAR_QBin=1 1467 578 434 336 262 196
CAR_QBin=2 1405 463 312 240 195 148
CAR_QBin=3 1418 422 279 206 152 110
HRR_QBin=1 1430 449 280 209 156 119
HRR_QBin=2 1430 500 348 259 207 154
HRR_QBin=3 1430 514 397 314 246 181
Joint_analysis=1 1314 522 398 311 242 181
Joint_analysis=2 368 135 90 66 54 40
Joint_analysis=3 1750 568 397 302 239 178
Joint_analysis=4 858 238 140 103 74 55
CAR_QBin=1 1207 675 576 498 453 416
CAR_QBin=2 1204 597 467 417 371 326
CAR_QBin=3 1195 527 383 307 265 230
HRR_QBin=1 1202 515 369 292 248 202
HRR_QBin=2 1203 620 490 428 379 347
HRR_QBin=3 1201 664 567 502 462 423
Joint_analysis=1 1578 883 745 661 602 557
Joint_analysis=2 189 76 56 44 40 32
Joint_analysis=3 1548 726 558 465 404 350
Joint_analysis=4 291 114 67 52 43 33
CAR_QBin=1 7071 2 0 0 0 0
CAR_QBin=2 7058 3 0 0 0 0
CAR_QBin=3 7062 5 1 1 1 1
HRR_QBin=1 7065 4 1 1 1 1
HRR_QBin=2 7062 6 0 0 0 0
HRR_QBin=3 7064 0 0 0 0 0
Joint_analysis=1 8169 3 0 0 0 0
Joint_analysis=2 662 0 0 0 0 0
Joint_analysis=3 10506 6 1 1 1 1
Joint_analysis=4 1854 1 0 0 0 0

Figure S3. Kaplan-Meier Curves for 1-Year All-Cause Mortality Stratified by CAR, HRR, and Their Combined Effects in MIMIC-IV, MIMIC-III, and eICU-CRD Cohorts

Notes: Panels show Kaplan–Meier curves for 1-Year all-cause mortality in critical heart failure across three cohorts; rows (top→bottom) are MIMIC-IV, MIMIC-III, and eICU-CRD, and columns (left→right) are CAR, HRR, and their joint analysis. In the joint analysis, groups 1–4 denote, respectively, High HRR & Low CAR, Low HRR & Low CAR, High HRR & High CAR, and Low HRR & High CAR. Log-rank *p*-values indicate between-group differences, and the numbers at risk at each time point are shown beneath each panel.

Abbreviations: CAR, creatinine-to-albumin ratio; HRR, hemoglobin-to-red cell distribution width ratio; KM, Kaplan-Meier; ICU, intensive care unit; MIMIC-IV/MIMIC-III, Medical Information Mart for Intensive Care IV/III; eICU-CRD, eICU Collaborative Research Database.

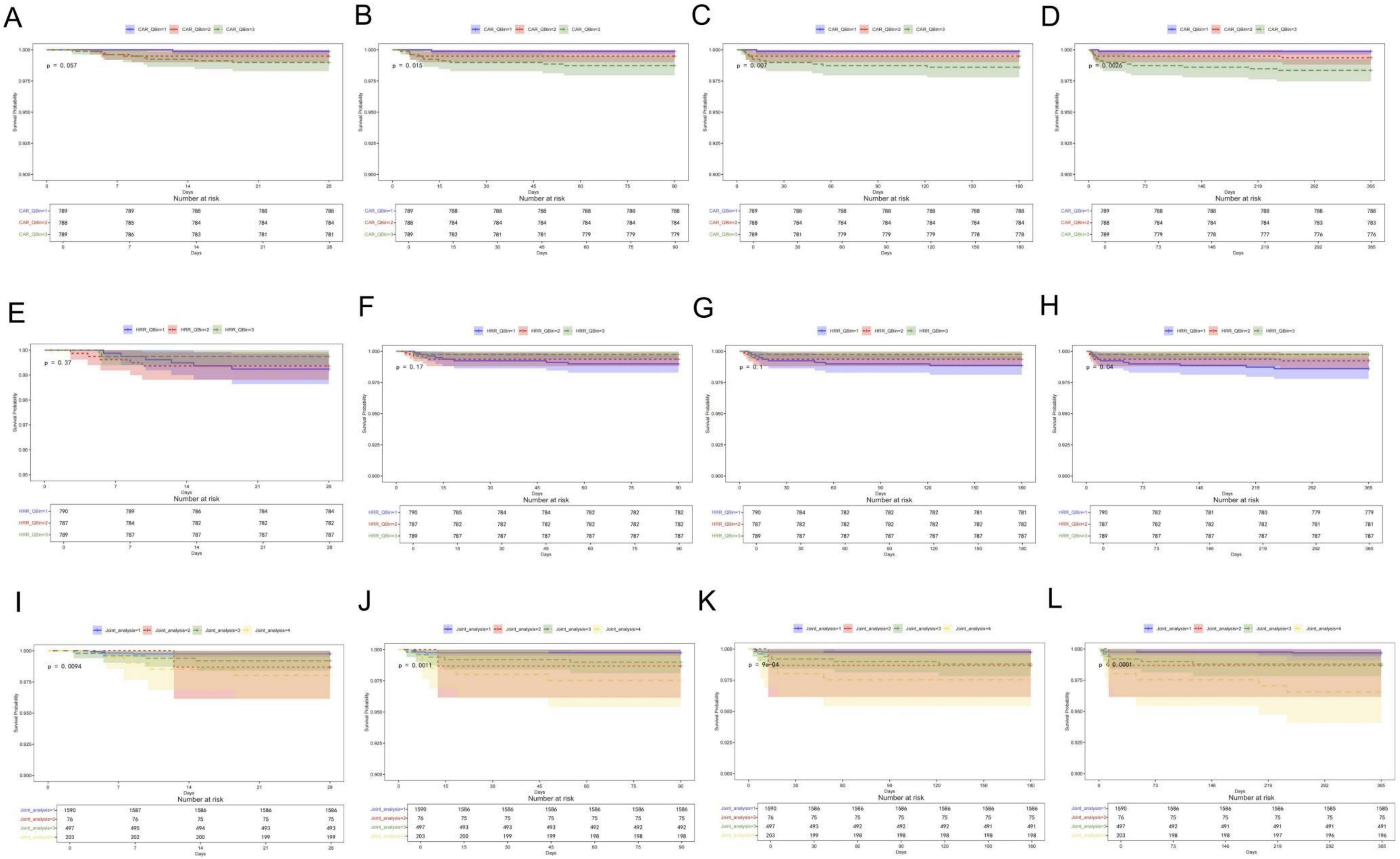
A
CAR_QBin=1 CAR_QBin=2 CAR_QBin=3
p = 0.057
B
p = 0.015
C
p = 0.007
D
p = 0.0026
E
HRR_QBin=1 HRR_QBin=2 HRR_QBin=3
p = 0.37
F
p = 0.17
G
p = 0.1
H
p = 0.04
I
Joint_analysis=1 Joint_analysis=2 Joint_analysis=3 Joint_analysis=4
p = 0.0094
J
p = 0.0011
K
p = 9e-04
L
p < 0.0001
Survival Probability
Days
Number at risk

Figure S4. Kaplan-Meier Curves for All-Cause Mortality Stratified by CAR, HRR, and Their Combined Effects in HOSP-CCU Cohort

Notes: Panels A–D display Kaplan–Meier survival curves stratified by tertiles of CAR; panels E–H show curves stratified by tertiles of HRR; panels I–L present the joint analysis of CAR and HRR (combined strata). The four columns, from left to right, correspond to 28-day, 90-day, 180-day, and 1-year all-cause mortality. Within each panel, log-rank *p*-values test differences between groups, and the number at risk is reported beneath each plot.

Abbreviations: CAR, creatinine-to-albumin ratio; HRR, hemoglobin-to-red cell distribution width ratio; KM, Kaplan-Meier; HOSP-CCU, Coronary Care Unit of the First Affiliated Hospital of Wenzhou Medical University.

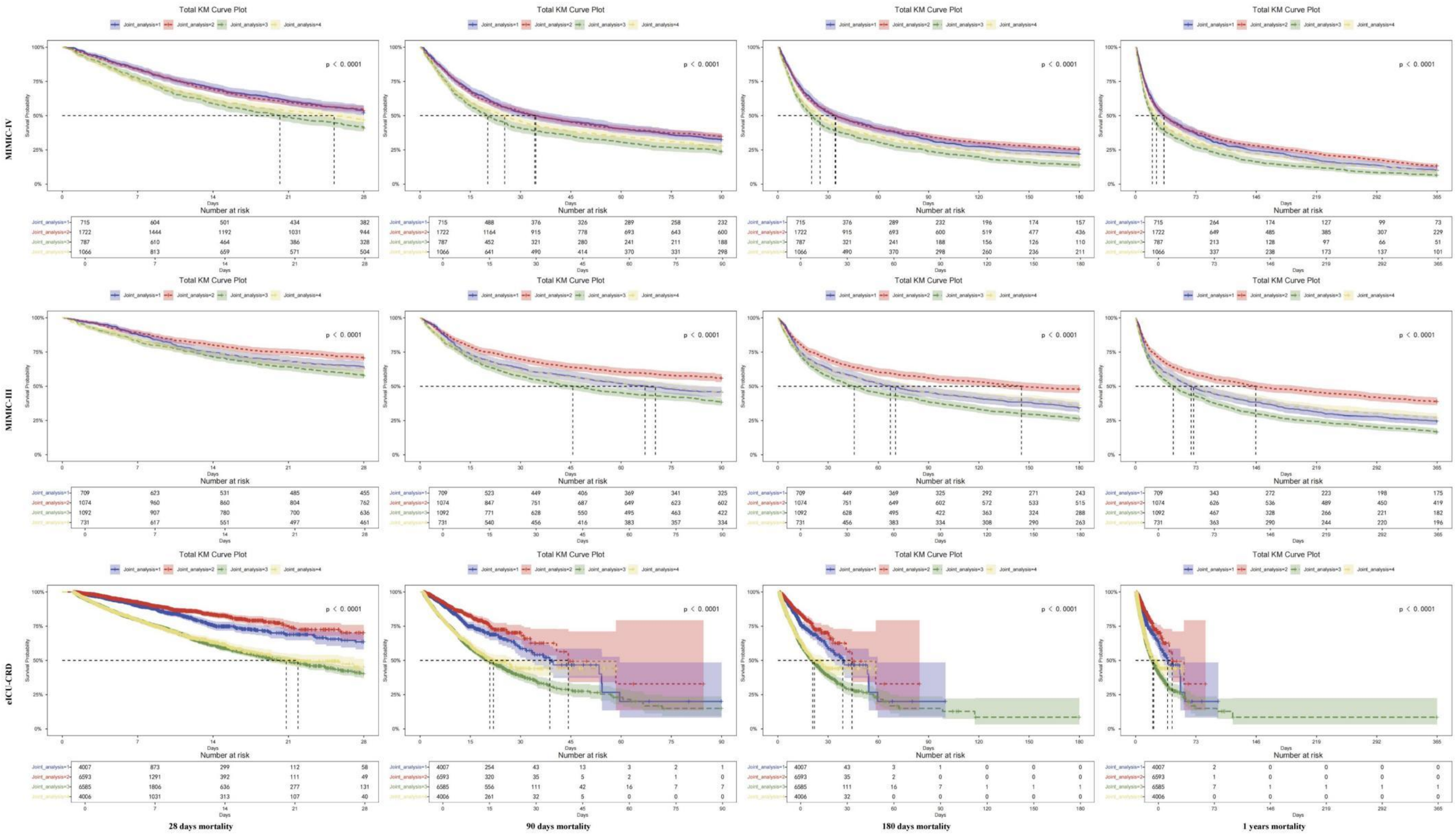
Total KM Curve Plot
Joint_analysis=1 Joint_analysis=2 Joint_analysis=3 Joint_analysis=4
p < 0.0001
Survival Probability
Days
Number at risk
MIMIC-IV
715 604 501 434 382
1722 1444 1192 1031 944
787 610 464 386 328
1066 813 659 571 504
715 488 376 326 289 258 232
1722 1164 915 778 693 643 600
787 452 321 280 241 211 188
1066 641 490 414 370 331 298
715 376 289 232 196 174 157
1722 915 693 600 519 477 436
787 321 241 188 156 126 110
1066 490 370 298 260 236 211
715 264 174 127 99 73
1722 649 485 385 307 229
787 213 128 97 66 51
1066 337 238 173 137 101
MIMIC-III
709 623 531 485 455
1074 960 860 804 762
1092 907 780 700 636
731 617 551 497 461
709 523 449 406 369 341 325
1074 847 751 687 649 623 602
1092 771 628 550 495 463 422
731 540 456 416 383 357 334
709 449 369 325 292 271 243
1074 751 649 602 572 533 515
1092 628 495 422 363 324 288
731 456 383 334 308 290 263
709 343 272 223 198 175
1074 626 536 489 450 419
1092 467 328 266 221 182
731 363 290 244 220 196
eICU-CRD
4007 873 299 112 58
6593 1291 392 111 49
6585 1806 636 277 131
4006 1031 313 107 40
4007 254 43 13 3 2 1
6593 320 35 5 2 1 0
6585 556 111 42 16 7 7
4006 261 32 5 0 0 0
4007 43 3 1 0 0 0
6593 35 2 0 0 0 0
6585 111 16 7 1 1 1
4006 32 0 0 0 0 0
4007 2 0 0 0 0
6593 1 0 0 0 0
6585 7 1 1 1 1
4006 0 0 0 0 0
28 days mortality
90 days mortality
180 days mortality
1 years mortality

Figure S5. Sensitivity Analysis of Kaplan-Meier Curves for All-Cause Mortality Stratified by CAR and HRR in MIMIC-IV, MIMIC-III, and eICU-CRD Cohorts

Notes: Panels display Kaplan–Meier survival curves with numbers-at-risk tables. Rows (top to bottom) correspond to the MIMIC-IV, MIMIC-III, and eICU-CRD cohorts; columns (left to right) correspond to 28-day, 90-day, 180-day, and 1-year all-cause mortality. The joint analysis dichotomizes HRR and CAR at their median values and combines them into four groups: 1 = High HRR & Low CAR; 2 = Low HRR & Low CAR; 3 = High HRR & High CAR; 4 = Low HRR & High CAR. Log-rank *p*-values indicate between-group differences, and the numbers at risk at each time point are shown beneath each panel.

Abbreviations: CAR, creatinine-to-albumin ratio; HRR, hemoglobin-to-red cell distribution width ratio; KM, Kaplan-Meier; ICU, intensive care unit; MIMIC-IV/MIMIC-III, Medical Information Mart for Intensive Care IV/III; eICU-CRD, eICU Collaborative Research Database.

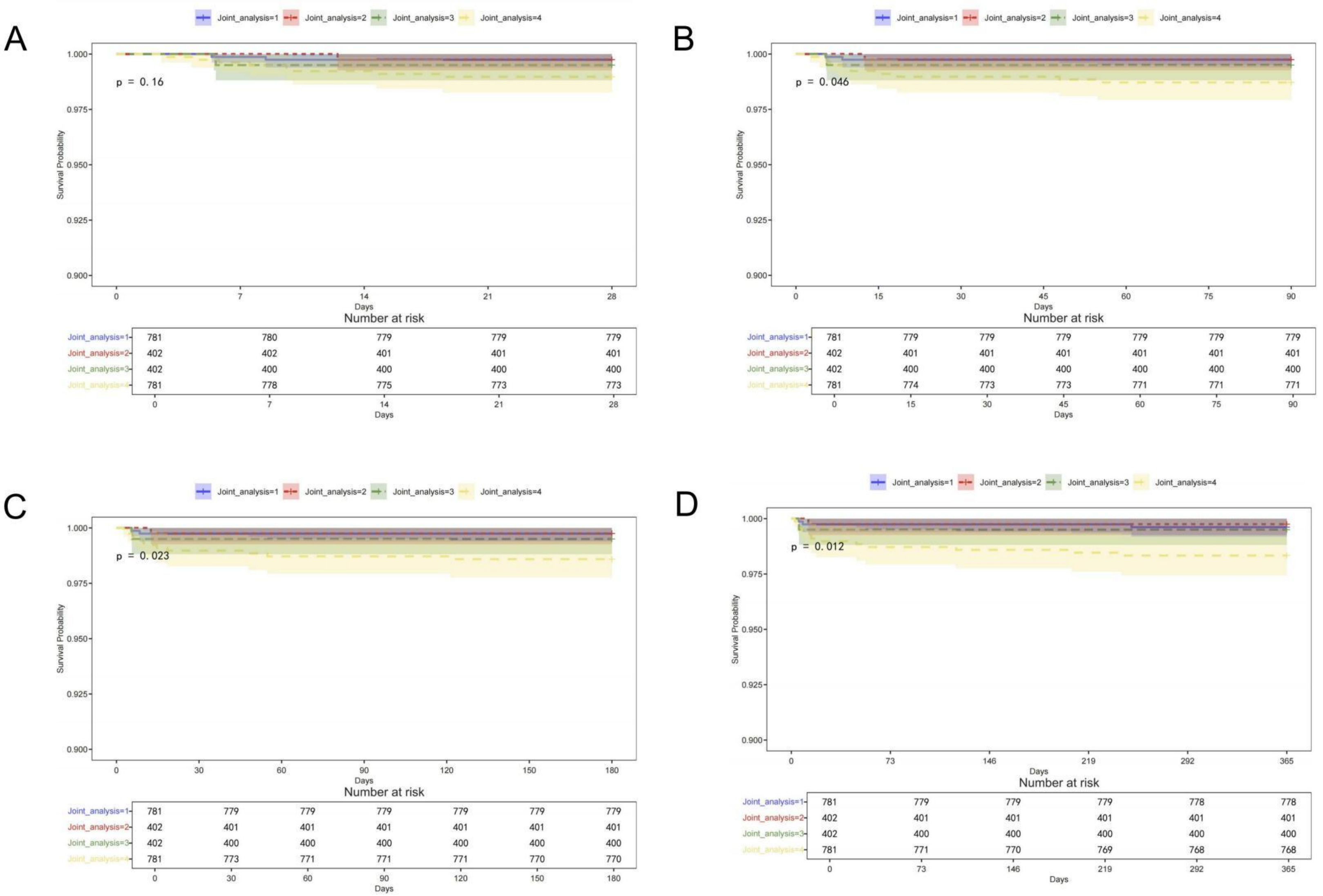

A
Joint_analysis=1 Joint_analysis=2 Joint_analysis=3 Joint_analysis=4
Survival Probability
p = 0.16
Days
Number at risk
Joint_analysis=1 781 780 779 779 779
Joint_analysis=2 402 402 401 401 401
Joint_analysis=3 402 400 400 400 400
Joint_analysis=4 781 778 775 773 773
0 7 14 21 28
Days
B
Joint_analysis=1 Joint_analysis=2 Joint_analysis=3 Joint_analysis=4
Survival Probability
p = 0.046
Days
Number at risk
Joint_analysis=1 781 779 779 779 779 779 779
Joint_analysis=2 402 401 401 401 401 401 401
Joint_analysis=3 402 400 400 400 400 400 400
Joint_analysis=4 781 774 773 773 771 771 771
0 15 30 45 60 75 90
Days
C
Joint_analysis=1 Joint_analysis=2 Joint_analysis=3 Joint_analysis=4
Survival Probability
p = 0.023
Days
Number at risk
Joint_analysis=1 781 779 779 779 779 779 779
Joint_analysis=2 402 401 401 401 401 401 401
Joint_analysis=3 402 400 400 400 400 400 400
Joint_analysis=4 781 773 771 771 771 770 770
0 30 60 90 120 150 180
Days
D
Joint_analysis=1 Joint_analysis=2 Joint_analysis=3 Joint_analysis=4
Survival Probability
p = 0.012
Days
Number at risk
Joint_analysis=1 781 779 779 779 778 778
Joint_analysis=2 402 401 401 401 401 401
Joint_analysis=3 402 400 400 400 400 400
Joint_analysis=4 781 771 770 769 768 768
0 73 146 219 292 365
Days

Figure S6. Sensitivity Analysis of Kaplan-Meier Curves for All-Cause Mortality Stratified by CAR, HRR, and Their Combined Effects in HOSP-CCU Cohort

Notes: Panels A–D correspond to 28-day, 90-day, 180-day, and 1-year all-cause mortality, respectively. The joint analysis dichotomizes HRR and CAR at their median values and combines them into four groups: 1 = High HRR & Low CAR; 2 = Low HRR & Low CAR; 3 = High HRR & High CAR; 4 = Low HRR & High CAR. Curves display Kaplan–Meier estimates; log-rank *p*-values test between-group differences, and numbers at risk are shown beneath each panel.

Abbreviations: CAR, creatinine-to-albumin ratio; HRR, hemoglobin-to-red cell distribution width ratio; KM, Kaplan-Meier; HOSP-CCU, Coronary Care Unit of the First Affiliated Hospital of Wenzhou Medical University.

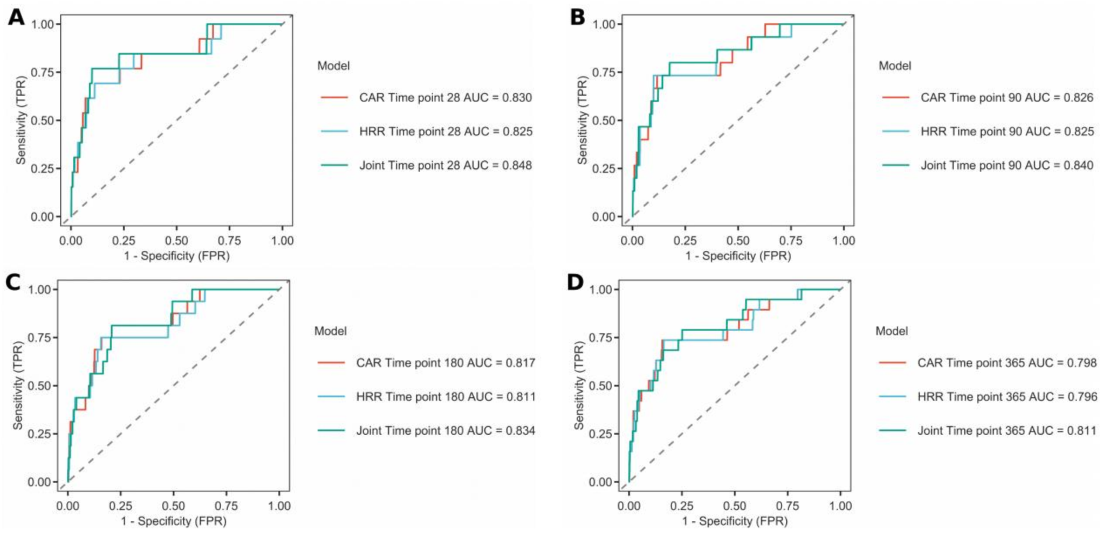

Figure S7. ROAUC curves for CAR, HRR, and their combination in predicting all-cause mortality at 28 days, 90 days, 180 days, and 1 year in the HOSP-CCU cohort

Notes: (A) 28-day all-cause mortality; (B) 90-day all-cause mortality; (C) 180-day all-cause mortality; (D) 1-year all-cause mortality in the HOSP-CCU cohort.

Abbreviations: HRR, hemoglobin-to-red cell distribution width ratio; CAR, creatinine-to-albumin ratio; AUC, area under the curve; HOSP-CCU, Coronary Care Unit of the First Affiliated Hospital of Wenzhou Medical University.

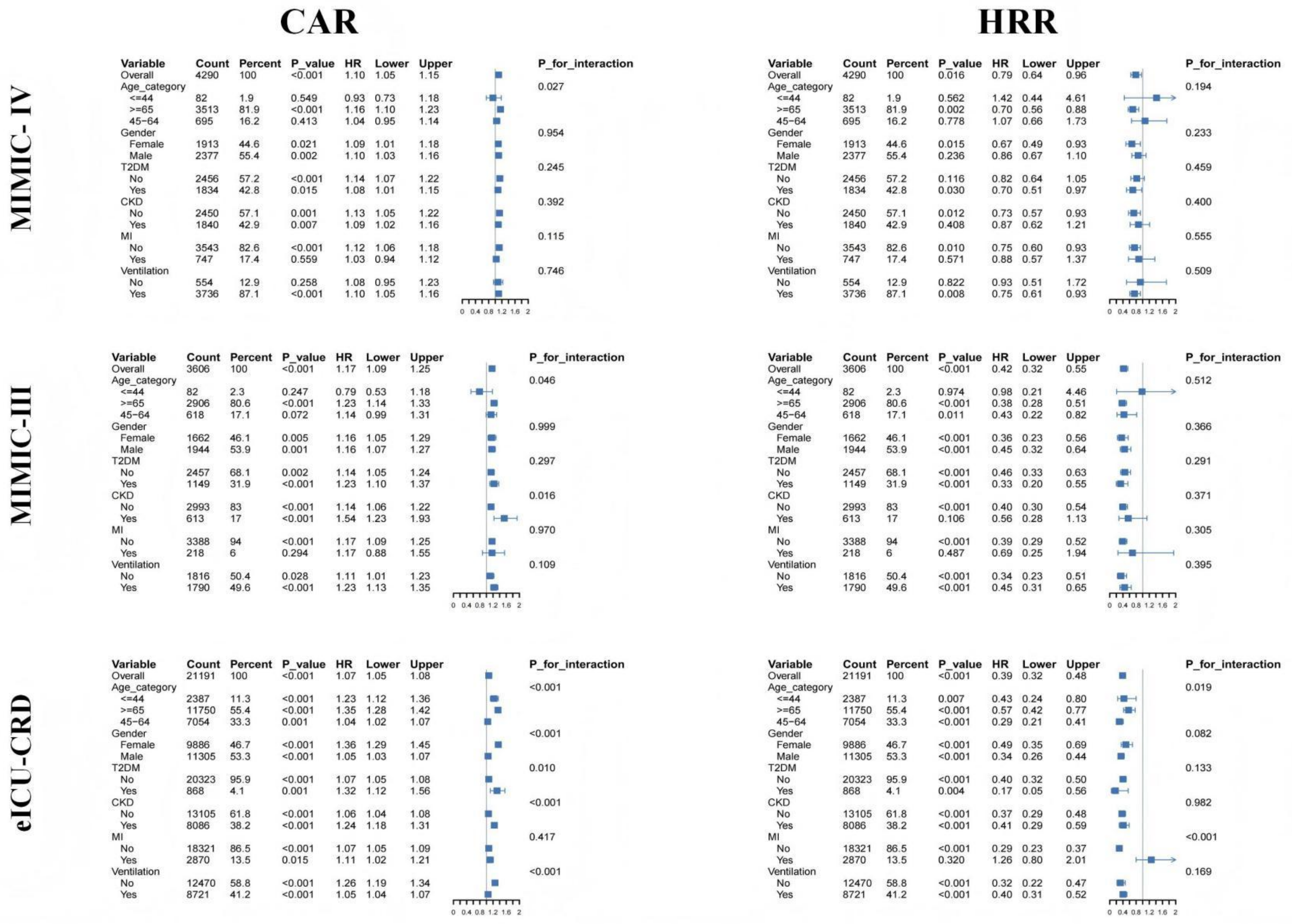
CAR
HRR
MIMIC- IV
Variable Count Percent P_value HR Lower Upper P_for_interaction
Overall 4290 100 <0.001 1.10 1.05 1.15
Age_category 0.027
<=44 82 1.9 0.549 0.93 0.73 1.18
>=65 3513 81.9 <0.001 1.16 1.10 1.23
45-64 695 16.2 0.413 1.04 0.95 1.14
Gender 0.954
Female 1913 44.6 0.021 1.09 1.01 1.18
Male 2377 55.4 0.002 1.10 1.03 1.16
T2DM 0.245
No 2456 57.2 <0.001 1.14 1.07 1.22
Yes 1834 42.8 0.015 1.08 1.01 1.15
CKD 0.392
No 2450 57.1 0.001 1.13 1.05 1.22
Yes 1840 42.9 0.007 1.09 1.02 1.16
MI 0.115
No 3543 82.6 <0.001 1.12 1.06 1.18
Yes 747 17.4 0.559 1.03 0.94 1.12
Ventilation 0.746
No 554 12.9 0.258 1.08 0.95 1.23
Yes 3736 87.1 <0.001 1.10 1.05 1.16
0 0.4 0.8 1.2 1.6 2
Variable Count Percent P_value HR Lower Upper P_for_interaction
Overall 4290 100 0.016 0.79 0.64 0.96
Age_category 0.194
<=44 82 1.9 0.562 1.42 0.44 4.61
>=65 3513 81.9 0.002 0.70 0.56 0.88
45-64 695 16.2 0.778 1.07 0.66 1.73
Gender 0.233
Female 1913 44.6 0.015 0.67 0.49 0.93
Male 2377 55.4 0.236 0.86 0.67 1.10
T2DM 0.459
No 2456 57.2 0.116 0.82 0.64 1.05
Yes 1834 42.8 0.030 0.70 0.51 0.97
CKD 0.400
No 2450 57.1 0.012 0.73 0.57 0.93
Yes 1840 42.9 0.408 0.87 0.62 1.21
MI 0.555
No 3543 82.6 0.010 0.75 0.60 0.93
Yes 747 17.4 0.571 0.88 0.57 1.37
Ventilation 0.509
No 554 12.9 0.822 0.93 0.51 1.72
Yes 3736 87.1 0.008 0.75 0.61 0.93
0 0.4 0.8 1.2 1.6 2
MIMIC-III
Variable Count Percent P_value HR Lower Upper P_for_interaction
Overall 3606 100 <0.001 1.17 1.09 1.25
Age_category 0.046
<=44 82 2.3 0.247 0.79 0.53 1.18
>=65 2906 80.6 <0.001 1.23 1.14 1.33
45-64 618 17.1 0.072 1.14 0.99 1.31
Gender 0.999
Female 1662 46.1 0.005 1.16 1.05 1.29
Male 1944 53.9 0.001 1.16 1.07 1.27
T2DM 0.297
No 2457 68.1 0.002 1.14 1.05 1.24
Yes 1149 31.9 <0.001 1.23 1.10 1.37
CKD 0.016
No 2993 83 <0.001 1.14 1.06 1.22
Yes 613 17 <0.001 1.54 1.23 1.93
MI 0.970
No 3388 94 <0.001 1.17 1.09 1.25
Yes 218 6 0.294 1.17 0.88 1.55
Ventilation 0.109
No 1816 50.4 0.028 1.11 1.01 1.23
Yes 1790 49.6 <0.001 1.23 1.13 1.35
0 0.4 0.8 1.2 1.6 2
Variable Count Percent P_value HR Lower Upper P_for_interaction
Overall 3606 100 <0.001 0.42 0.32 0.55
Age_category 0.512
<=44 82 2.3 0.974 0.98 0.21 4.46
>=65 2906 80.6 <0.001 0.38 0.28 0.51
45-64 618 17.1 0.011 0.43 0.22 0.82
Gender 0.366
Female 1662 46.1 <0.001 0.36 0.23 0.56
Male 1944 53.9 <0.001 0.45 0.32 0.64
T2DM 0.291
No 2457 68.1 <0.001 0.46 0.33 0.63
Yes 1149 31.9 <0.001 0.33 0.20 0.55
CKD 0.371
No 2993 83 <0.001 0.40 0.30 0.54
Yes 613 17 0.106 0.56 0.28 1.13
MI 0.305
No 3388 94 <0.001 0.39 0.29 0.52
Yes 218 6 0.487 0.69 0.25 1.94
Ventilation 0.395
No 1816 50.4 <0.001 0.34 0.23 0.51
Yes 1790 49.6 <0.001 0.45 0.31 0.65
0 0.4 0.8 1.2 1.6 2
eICU-CRD
Variable Count Percent P_value HR Lower Upper P_for_interaction
Overall 21191 100 <0.001 1.07 1.05 1.08
Age_category <0.001
<=44 2387 11.3 <0.001 1.23 1.12 1.36
>=65 11750 55.4 <0.001 1.35 1.28 1.42
45-64 7054 33.3 0.001 1.04 1.02 1.07
Gender <0.001
Female 9886 46.7 <0.001 1.36 1.29 1.45
Male 11305 53.3 <0.001 1.05 1.03 1.07
T2DM 0.010
No 20323 95.9 <0.001 1.07 1.05 1.08
Yes 868 4.1 0.001 1.32 1.12 1.56
CKD <0.001
No 13105 61.8 <0.001 1.06 1.04 1.08
Yes 8086 38.2 <0.001 1.24 1.18 1.31
MI 0.417
No 18321 86.5 <0.001 1.07 1.05 1.09
Yes 2870 13.5 0.015 1.11 1.02 1.21
Ventilation <0.001
No 12470 58.8 <0.001 1.26 1.19 1.34
Yes 8721 41.2 <0.001 1.05 1.04 1.07
0 0.4 0.8 1.2 1.6 2
Variable Count Percent P_value HR Lower Upper P_for_interaction
Overall 21191 100 <0.001 0.39 0.32 0.48
Age_category 0.019
<=44 2387 11.3 0.007 0.43 0.24 0.80
>=65 11750 55.4 <0.001 0.57 0.42 0.77
45-64 7054 33.3 <0.001 0.29 0.21 0.41
Gender 0.082
Female 9886 46.7 <0.001 0.49 0.35 0.69
Male 11305 53.3 <0.001 0.34 0.26 0.44
T2DM 0.133
No 20323 95.9 <0.001 0.40 0.32 0.50
Yes 868 4.1 0.004 0.17 0.05 0.56
CKD 0.982
No 13105 61.8 <0.001 0.37 0.29 0.48
Yes 8086 38.2 <0.001 0.41 0.29 0.59
MI <0.001
No 18321 86.5 <0.001 0.29 0.23 0.37
Yes 2870 13.5 0.320 1.26 0.80 2.01
Ventilation 0.169
No 12470 58.8 <0.001 0.32 0.22 0.47
Yes 8721 41.2 <0.001 0.40 0.31 0.52
0 0.4 0.8 1.2 1.6 2

Figure S8. Subgroup Analysis of 90-Day All-Cause Mortality Stratified by CAR and HRR in MIMIC-IV, MIMIC-III, and eICU-CRD Cohorts

Notes: The figure presents subgroup analyses for 90-day all-cause mortality according to CAR (left panels) and HRR (right panels) in the MIMIC-IV, MIMIC-III, and eICU-CRD cohorts. Hazard ratios (HRs) and 95% confidence intervals (CIs) were estimated using Cox proportional hazards models. *P* values for interaction are shown for each subgroup variable.

Abbreviations: HR, hazard ratio; CI, confidence interval; HRR, hemoglobin-to-red cell distribution width ratio; CAR, creatinine-to-albumin ratio; T2DM, type 2 diabetes mellitus; CKD, chronic kidney disease; MI, myocardial infarction; MIMIC-IV/MIMIC-III, Medical Information Mart for Intensive Care IV/III; eICU-CRD, eICU Collaborative Research Database.

CAR

MIMIC- IV

Variable	Count	Percent	P_value	HR	Lower	Upper	P_for_interaction
Overall	4290	100	<0.001	1.09	1.05	1.14	
Age_category							0.026
<=44	82	1.9	0.375	0.90	0.70	1.14	
>=65	3513	81.9	<0.001	1.15	1.09	1.21	
45-64	695	16.2	0.263	1.05	0.96	1.14	
Gender							0.853
Female	1913	44.6	0.012	1.09	1.02	1.17	
Male	2377	55.4	0.003	1.09	1.03	1.15	
T2DM							0.319
No	2456	57.2	<0.001	1.13	1.06	1.20	
Yes	1834	42.8	0.012	1.08	1.02	1.14	
CKD							0.476
No	2450	57.1	0.002	1.12	1.04	1.20	
Yes	1840	42.9	0.007	1.08	1.02	1.15	
MI							0.300
No	3543	82.6	<0.001	1.10	1.05	1.16	
Yes	747	17.4	0.275	1.05	0.96	1.13	
Ventilation							0.324
No	554	12.9	0.595	1.03	0.91	1.17	
Yes	3736	87.1	<0.001	1.10	1.06	1.16	

0 0.4 0.8 1.2 1.6 2

MIMIC-III

Variable	Count	Percent	P_value	HR	Lower	Upper	P_for_interaction
Overall	3606	100	<0.001	1.18	1.11	1.25	
Age_category							0.030
<=44	82	2.3	0.362	0.85	0.59	1.21	
>=65	2906	80.6	<0.001	1.25	1.16	1.34	
45-64	618	17.1	0.091	1.12	0.98	1.27	
Gender							0.703
Female	1662	46.1	<0.001	1.19	1.08	1.30	
Male	1944	53.9	<0.001	1.16	1.07	1.26	
T2DM							0.100
No	2457	68.1	0.001	1.14	1.06	1.23	
Yes	1149	31.9	<0.001	1.27	1.15	1.4	
CKD							0.013
No	2993	83	<0.001	1.15	1.08	1.22	
Yes	613	17	<0.001	1.55	1.26	1.90	
MI							0.912
No	3388	94	<0.001	1.18	1.11	1.25	
Yes	218	6	0.304	1.15	0.88	1.51	
Ventilation							0.067
No	1816	50.4	0.008	1.12	1.03	1.23	
Yes	1790	49.6	<0.001	1.25	1.15	1.36	

0 0.4 0.8 1.2 1.6 2

eICU-CRD

Variable	Count	Percent	P_value	HR	Lower	Upper	P_for_interaction
Overall	21191	100	<0.001	1.07	1.05	1.08	
Age_category							<0.001
<=44	2387	11.3	<0.001	1.23	1.12	1.36	
>=65	11750	55.4	<0.001	1.35	1.28	1.42	
45-64	7054	33.3	0.001	1.04	1.02	1.07	
Gender							<0.001
Female	9886	46.7	<0.001	1.36	1.29	1.45	
Male	11305	53.3	<0.001	1.05	1.03	1.07	
T2DM							0.010
No	20323	95.9	<0.001	1.07	1.05	1.08	
Yes	868	4.1	0.001	1.32	1.12	1.56	
CKD							<0.001
No	13105	61.8	<0.001	1.06	1.04	1.08	
Yes	8086	38.2	<0.001	1.24	1.18	1.31	
MI							0.416
No	18321	86.5	<0.001	1.07	1.05	1.09	
Yes	2870	13.5	0.015	1.11	1.02	1.21	
Ventilation							<0.001
No	12470	58.8	<0.001	1.26	1.19	1.34	
Yes	8721	41.2	<0.001	1.05	1.04	1.07	

0 0.4 0.8 1.2 1.6 2

HRR

MIMIC- IV

Variable	Count	Percent	P_value	HR	Lower	Upper	P_for_interaction
Overall	4290	100	0.001	0.72	0.60	0.87	
Age_category							0.450
<=44	82	1.9	0.902	1.07	0.35	3.31	
>=65	3513	81.9	<0.001	0.67	0.54	0.82	
45-64	695	16.2	0.554	0.87	0.56	1.37	
Gender							0.170
Female	1913	44.6	0.001	0.61	0.45	0.82	
Male	2377	55.4	0.060	0.80	0.63	1.01	
T2DM							0.823
No	2456	57.2	0.007	0.72	0.57	0.91	
Yes	1834	42.8	0.015	0.69	0.51	0.93	
CKD							0.569
No	2450	57.1	0.002	0.69	0.55	0.87	
Yes	1840	42.9	0.093	0.76	0.56	1.05	
MI							0.237
No	3543	82.6	<0.001	0.67	0.54	0.82	
Yes	747	17.4	0.633	0.90	0.59	1.37	
Ventilation							0.226
No	554	12.9	0.915	0.97	0.56	1.69	
Yes	3736	87.1	<0.001	0.68	0.56	0.83	

0 0.4 0.8 1.2 1.6 2

MIMIC-III

Variable	Count	Percent	P_value	HR	Lower	Upper	P_for_interaction
Overall	3606	100	<0.001	0.34	0.27	0.44	
Age_category							0.535
<=44	82	2.3	0.647	0.70	0.16	3.17	
>=65	2906	80.6	<0.001	0.31	0.23	0.41	
45-64	618	17.1	0.001	0.37	0.21	0.66	
Gender							0.579
Female	1662	46.1	<0.001	0.32	0.21	0.47	
Male	1944	53.9	<0.001	0.36	0.26	0.49	
T2DM							0.139
No	2457	68.1	<0.001	0.38	0.29	0.52	
Yes	1149	31.9	<0.001	0.26	0.16	0.40	
CKD							0.376
No	2993	83	<0.001	0.34	0.26	0.44	
Yes	613	17	0.012	0.44	0.23	0.83	
MI							0.312
No	3388	94	<0.001	0.32	0.25	0.42	
Yes	218	6	0.223	0.55	0.21	1.44	
Ventilation							0.387
No	1816	50.4	<0.001	0.29	0.20	0.41	
Yes	1790	49.6	<0.001	0.37	0.26	0.51	

0 0.4 0.8 1.2 1.6 2

eICU-CRD

Variable	Count	Percent	P_value	HR	Lower	Upper	P_for_interaction
Overall	21191	100	<0.001	0.39	0.32	0.48	
Age_category							0.020
<=44	2387	11.3	0.007	0.43	0.24	0.80	
>=65	11750	55.4	<0.001	0.57	0.42	0.77	
45-64	7054	33.3	<0.001	0.29	0.21	0.41	
Gender							0.079
Female	9886	46.7	<0.001	0.50	0.35	0.70	
Male	11305	53.3	<0.001	0.34	0.26	0.44	
T2DM							0.132
No	20323	95.9	<0.001	0.40	0.32	0.50	
Yes	868	4.1	0.004	0.17	0.05	0.56	
CKD							0.986
No	13105	61.8	<0.001	0.37	0.29	0.48	
Yes	8086	38.2	<0.001	0.41	0.29	0.59	
MI							<0.001
No	18321	86.5	<0.001	0.29	0.23	0.37	
Yes	2870	13.5	0.320	1.26	0.80	2.01	
Ventilation							0.169
No	12470	58.8	<0.001	0.32	0.22	0.47	
Yes	8721	41.2	<0.001	0.40	0.31	0.52	

0 0.4 0.8 1.2 1.6 2

Figure S9. Subgroup Analysis of 180-Day All-Cause Mortality Stratified by CAR and HRR in MIMIC-IV, MIMIC-III, and eICU-CRD Cohorts

Notes: The figure presents subgroup analyses for 180-day all-cause mortality according to CAR (left panels) and HRR (right panels) in the MIMIC-IV, MIMIC-III, and eICU-CRD cohorts. Hazard ratios (HRs) and 95% confidence intervals (CIs) were estimated using Cox proportional hazards models. *P* values for interaction are shown for each subgroup variable.

Abbreviations: HR, hazard ratio; CI, confidence interval; HRR, hemoglobin-to-red cell distribution width ratio; CAR, creatinine-to-albumin ratio; T2DM, type 2 diabetes mellitus; CKD, chronic kidney disease; MI, myocardial infarction; MIMIC-IV/MIMIC-III, Medical Information Mart for Intensive Care IV/III; eICU-CRD, eICU Collaborative Research Database.

MIMIC- IV

CAR

Variable	Count	Percent	P_value	HR	Lower	Upper	P_for_interaction
Overall	4290	100	<0.001	1.10	1.05	1.14	
Age_category							0.001
<=44	82	1.9	0.249	0.87	0.69	1.10	
>=65	3513	81.9	<0.001	1.17	1.11	1.23	
45−64	695	16.2	0.470	1.03	0.95	1.12	
Gender							0.844
Female	1913	44.6	0.004	1.10	1.03	1.18	
Male	2377	55.4	0.001	1.09	1.04	1.15	
T2DM							0.700
No	2456	57.2	0.001	1.11	1.05	1.18	
Yes	1834	42.8	0.001	1.10	1.04	1.16	
CKD							0.522
No	2450	57.1	0.001	1.12	1.05	1.19	
Yes	1840	42.9	0.002	1.09	1.03	1.15	
MI							0.116
No	3543	82.6	<0.001	1.12	1.07	1.17	
Yes	747	17.4	0.373	1.04	0.96	1.12	
Ventilation							0.444
No	554	12.9	0.281	1.06	0.95	1.19	
Yes	3736	87.1	<0.001	1.11	1.06	1.16	

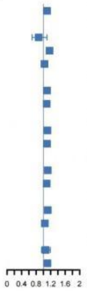

HRR

Variable	Count	Percent	P_value	HR	Lower	Upper	P_for_interaction
Overall	4290	100	<0.001	0.70	0.59	0.83	
Age_category							0.554
<=44	82	1.9	0.638	0.77	0.27	2.25	
>=65	3513	81.9	<0.001	0.66	0.54	0.80	
45−64	695	16.2	0.371	0.83	0.54	1.26	
Gender							0.052
Female	1913	44.6	<0.001	0.56	0.43	0.74	
Male	2377	55.4	0.058	0.8	0.64	1.01	
T2DM							0.825
No	2456	57.2	0.003	0.71	0.57	0.89	
Yes	1834	42.8	0.005	0.66	0.50	0.88	
CKD							0.348
No	2450	57.1	<0.001	0.67	0.54	0.83	
Yes	1840	42.9	0.086	0.77	0.57	1.04	
MI							0.172
No	3543	82.6	<0.001	0.65	0.53	0.79	
Yes	747	17.4	0.604	0.90	0.60	1.34	
Ventilation							0.498
No	554	12.9	0.356	0.79	0.47	1.31	
Yes	3736	87.1	<0.001	0.68	0.56	0.82	

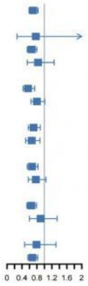

MIMIC-III

Variable	Count	Percent	P_value	HR	Lower	Upper	P_for_interaction
Overall	3606	100	<0.001	1.19	1.13	1.26	
Age_category							0.013
<=44	82	2.3	0.497	0.89	0.65	1.23	
>=65	2906	80.6	<0.001	1.27	1.19	1.36	
45−64	618	17.1	0.086	1.11	0.99	1.25	
Gender							0.723
Female	1662	46.1	<0.001	1.20	1.11	1.31	
Male	1944	53.9	<0.001	1.18	1.10	1.27	
T2DM							0.132
No	2457	68.1	<0.001	1.16	1.08	1.25	
Yes	1149	31.9	<0.001	1.28	1.16	1.40	
CKD							0.012
No	2993	83	<0.001	1.17	1.10	1.24	
Yes	613	17	<0.001	1.58	1.30	1.92	
MI							0.995
No	3388	94	<0.001	1.20	1.13	1.27	
Yes	218	6	0.187	1.18	0.92	1.52	
Ventilation							0.112
No	1816	50.4	<0.001	1.16	1.07	1.25	
Yes	1790	49.6	<0.001	1.25	1.16	1.36	

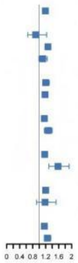

Variable	Count	Percent	P_value	HR	Lower	Upper	P_for_interaction
Overall	3606	100	<0.001	0.31	0.25	0.39	
Age_category							0.807
<=44	82	2.3	0.368	0.51	0.12	2.19	
>=65	2906	80.6	<0.001	0.30	0.23	0.39	
45−64	618	17.1	<0.001	0.28	0.16	0.48	
Gender							0.789
Female	1662	46.1	<0.001	0.33	0.23	0.47	
Male	1944	53.9	<0.001	0.30	0.22	0.40	
T2DM							0.124
No	2457	68.1	<0.001	0.35	0.27	0.46	
Yes	1149	31.9	<0.001	0.24	0.15	0.36	
CKD							0.357
No	2993	83	<0.001	0.31	0.24	0.40	
Yes	613	17	0.002	0.39	0.22	0.72	
MI							0.176
No	3388	94	<0.001	0.29	0.23	0.37	
Yes	218	6	0.219	0.57	0.23	1.40	
Ventilation							0.796
No	1816	50.4	<0.001	0.28	0.20	0.39	
Yes	1790	49.6	<0.001	0.32	0.23	0.44	

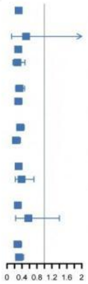

eICU-CRD

Variable	Count	Percent	P_value	HR	Lower	Upper	P_for_interaction
Overall	21191	100	<0.001	1.07	1.05	1.08	
Age_category							<0.001
<=44	2387	11.3	<0.001	1.23	1.12	1.36	
>=65	11750	55.4	<0.001	1.35	1.28	1.42	
45−64	7054	33.3	0.001	1.04	1.02	1.07	
Gender							<0.001
Female	9886	46.7	<0.001	1.36	1.29	1.45	
Male	11305	53.3	<0.001	1.05	1.03	1.07	
T2DM							0.010
No	20323	95.9	<0.001	1.07	1.05	1.08	
Yes	868	4.1	0.001	1.32	1.12	1.56	
CKD							<0.001
No	13105	61.8	<0.001	1.06	1.04	1.08	
Yes	8086	38.2	<0.001	1.24	1.18	1.31	
MI							0.416
No	18321	86.5	<0.001	1.07	1.05	1.09	
Yes	2870	13.5	0.015	1.11	1.02	1.21	
Ventilation							<0.001
No	12470	58.8	<0.001	1.26	1.19	1.34	
Yes	8721	41.2	<0.001	1.05	1.04	1.07	

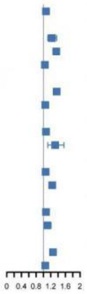

Variable	Count	Percent	P_value	HR	Lower	Upper	P_for_interaction
Overall	21191	100	<0.001	0.39	0.32	0.48	
Age_category							0.020
<=44	2387	11.3	0.007	0.43	0.24	0.80	
>=65	11750	55.4	<0.001	0.57	0.42	0.77	
45−64	7054	33.3	<0.001	0.29	0.21	0.41	
Gender							0.079
Female	9886	46.7	<0.001	0.50	0.35	0.70	
Male	11305	53.3	<0.001	0.34	0.26	0.44	
T2DM							0.132
No	20323	95.9	<0.001	0.40	0.32	0.50	
Yes	868	4.1	0.004	0.17	0.05	0.56	
CKD							0.986
No	13105	61.8	<0.001	0.37	0.29	0.48	
Yes	8086	38.2	<0.001	0.41	0.29	0.59	
MI							<0.001
No	18321	86.5	<0.001	0.29	0.23	0.37	
Yes	2870	13.5	0.320	1.26	0.80	2.01	
Ventilation							0.169
No	12470	58.8	<0.001	0.32	0.22	0.47	
Yes	8721	41.2	<0.001	0.40	0.31	0.52	

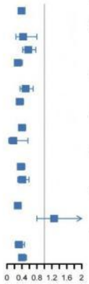

Figure S10. Subgroup Analysis of 1-Year All-Cause Mortality Stratified by CAR and HRR in MIMIC-IV, MIMIC-III, and eICU-CRD Cohorts

Notes: The figure presents subgroup analyses for 1-Year all-cause mortality according to CAR (left panels) and HRR (right panels) in the MIMIC-IV, MIMIC-III, and eICU-CRD cohorts. Hazard ratios (HRs) and 95% confidence intervals (CIs) were estimated using Cox proportional hazards models. *P* values for interaction are shown for each subgroup variable.

Abbreviations: HR, hazard ratio; CI, confidence interval; HRR, hemoglobin-to-red cell distribution width ratio; CAR, creatinine-to-albumin ratio; T2DM, type 2 diabetes mellitus; CKD, chronic kidney disease; MI, myocardial infarction; MIMIC-IV/MIMIC-III, Medical Information Mart for Intensive Care IV/III; eICU-CRD, eICU Collaborative Research Database.

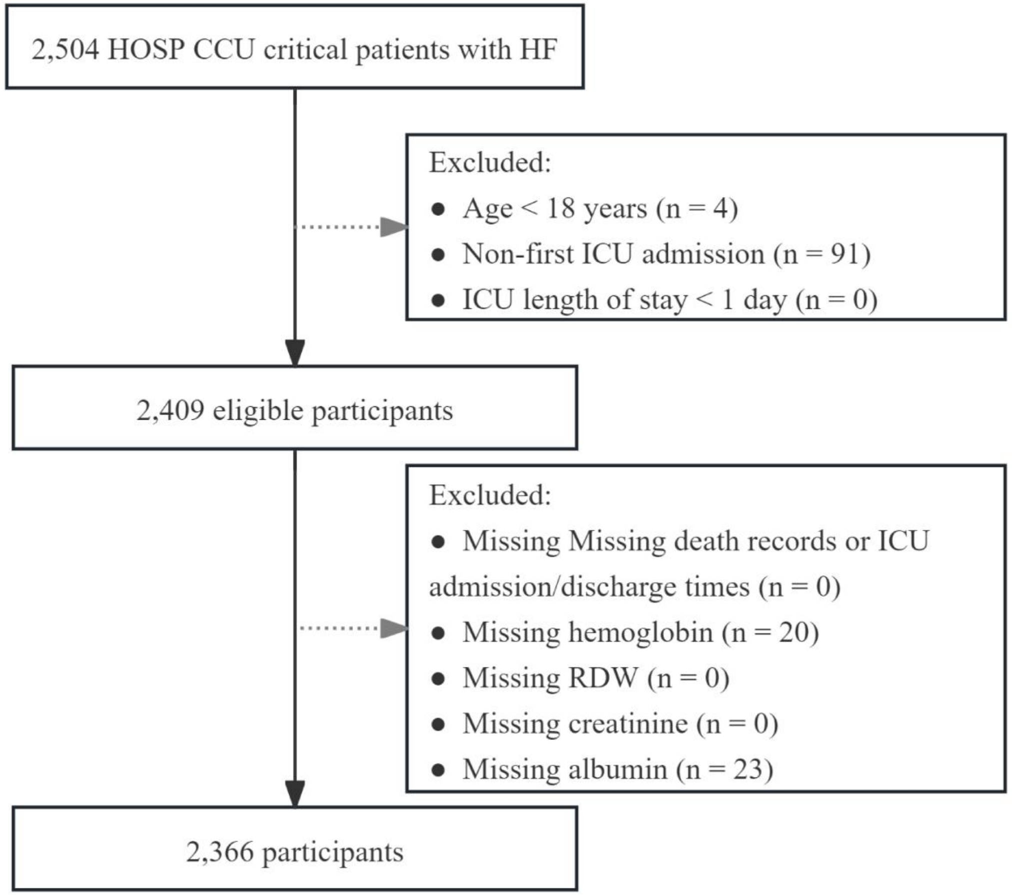

Figure S11. Flowchart of patient selection from HOSP-CCU databases for critical patients with heart failure

Abbreviations: HF, heart failure; HOSP-CCU, Coronary Care Unit of the First Affiliated Hospital of Wenzhou Medical University; ICU, intensive care unit; RDW, red cell distribution width.

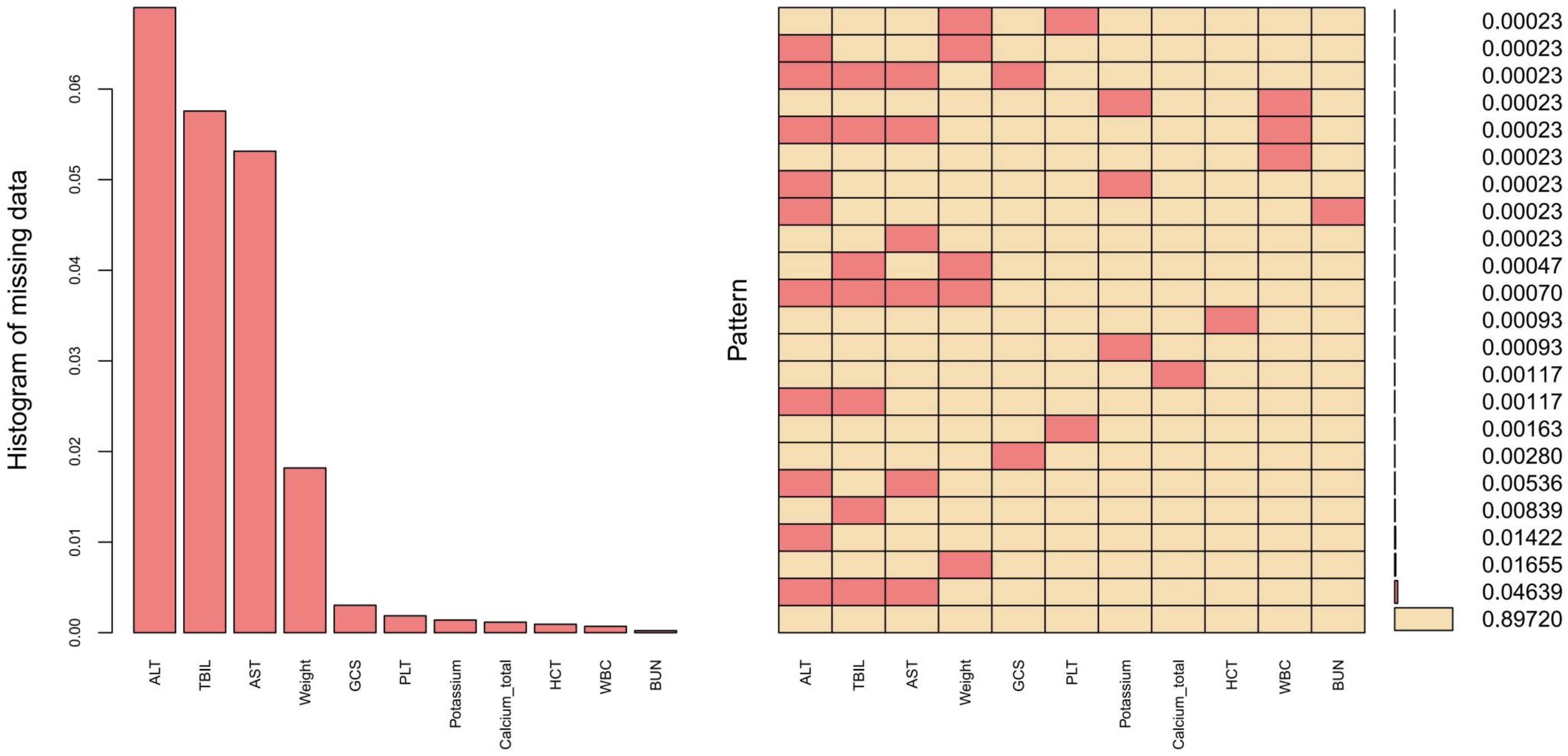

Figure S12. Missing Data Visualization of Covariates in the MIMIC-IV Database

Abbreviations: ALT: Alanine Aminotransferase; TBIL: Total Bilirubin; AST: Aspartate Aminotransferase; GCS: Glasgow Coma Scale; PLT: Platelets; HCT: Hematocrit; WBC: White Blood Cell Count; BUN: Blood Urea Nitrogen.

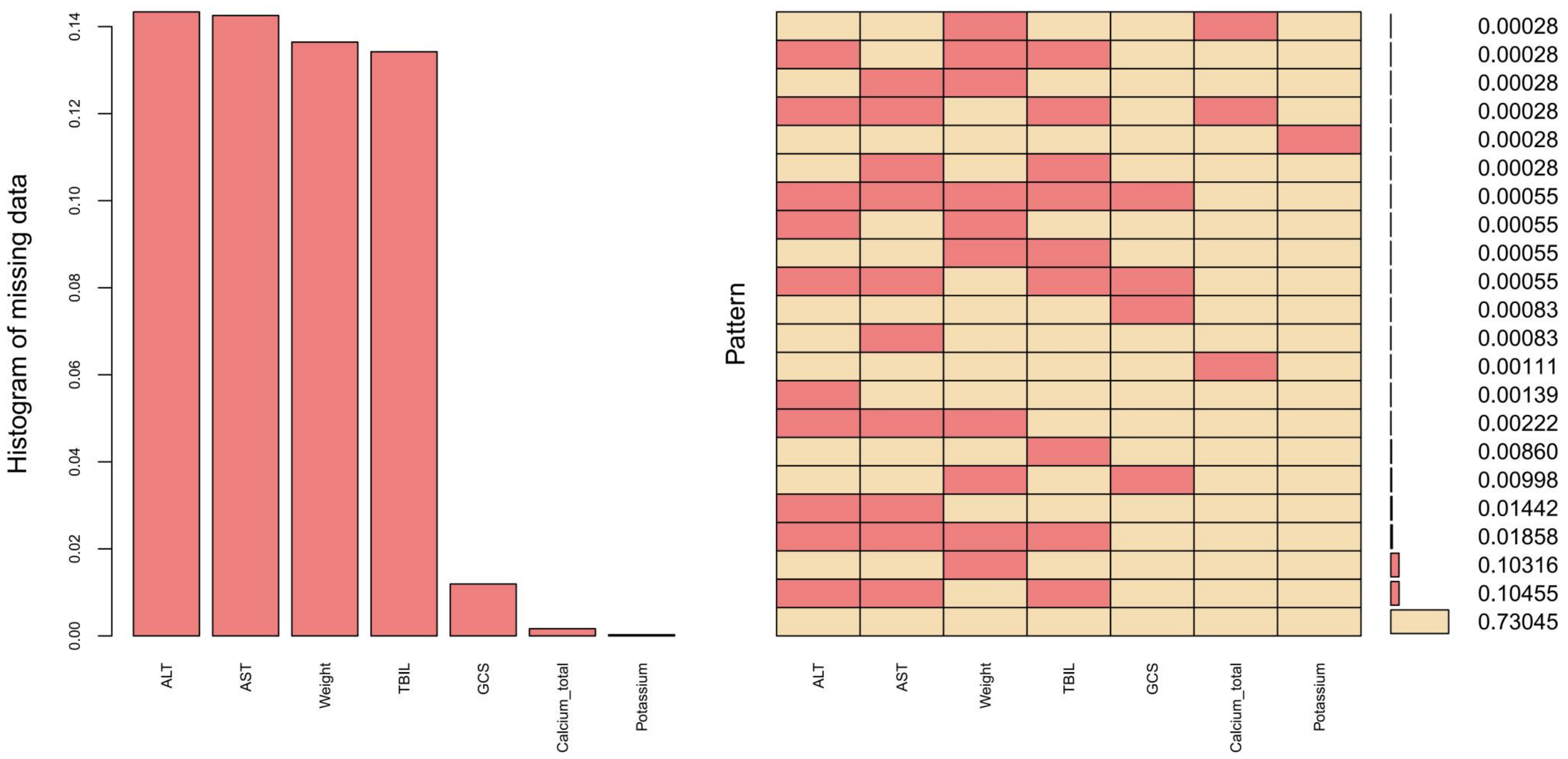

Figure S13. Missing Data Visualization of Covariates in the MIMIC-III Database

Abbreviations: ALT: Alanine Aminotransferase; TBIL: Total Bilirubin; AST: Aspartate Aminotransferase; GCS: Glasgow Coma Scale.

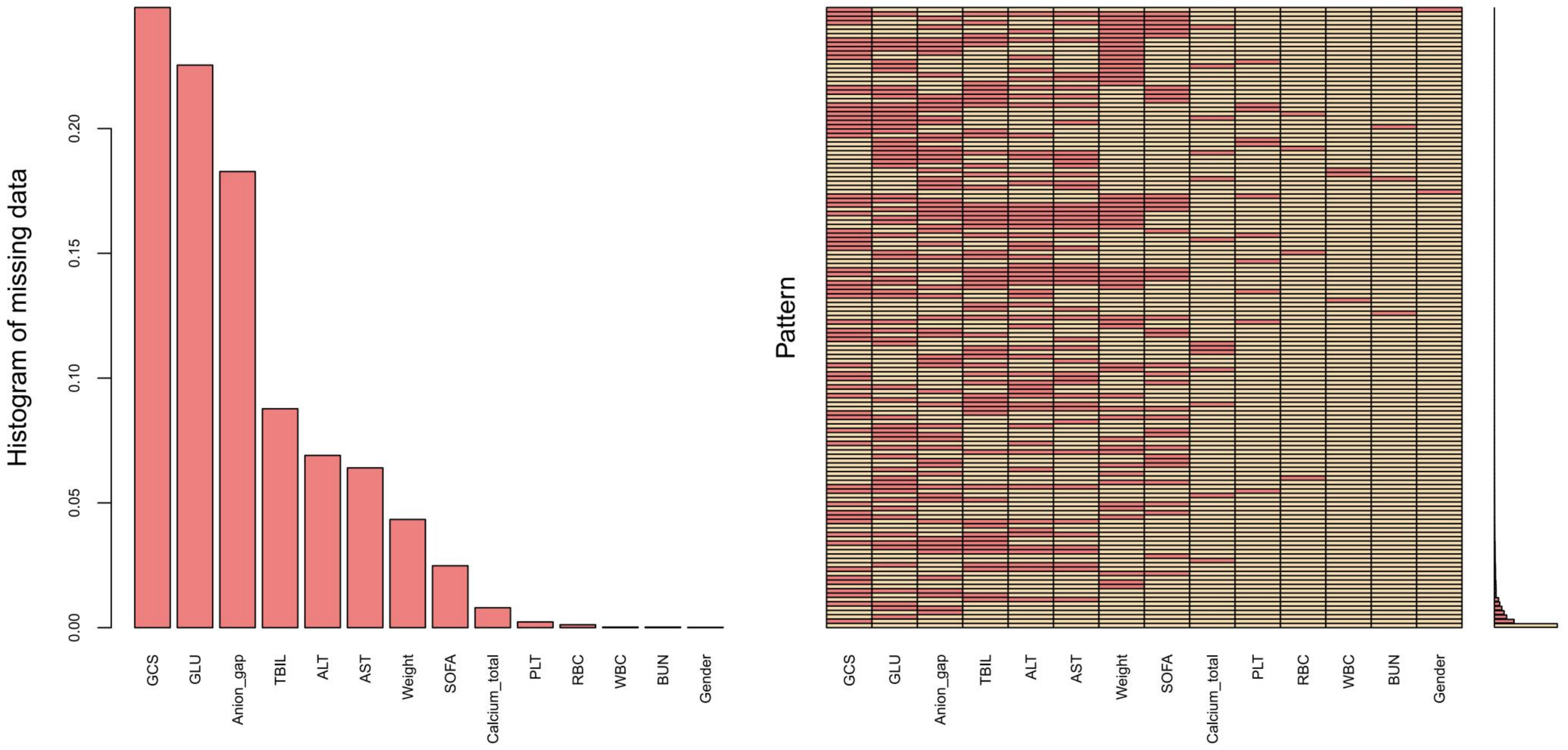

Figure S14. Missing Data Visualization of Covariates in the EICU-CRD Database

Abbreviations: GCS: Glasgow Coma Scale; GLU: Glucose; TBIL: Total Bilirubin; ALT: Alanine Aminotransferase; AST: Aspartate Aminotransferase; SOFA: Sequential Organ Failure Assessment; PLT: Platelets; RBC: Red Blood Cell Count; WBC: White Blood Cell Count;

BUN: Blood Urea Nitrogen.

Table S5. Association of HRR and CAR with 90-Day Mortality in Three critical ill Cohorts

Cohort	Exposure	Level	Model 1 HR (95% CI)	*p* value	Model 2 HR (95% CI)	*p* value	Model 3 HR (95% CI)	*p* value
**MIMIC-IV**	**HRR**	Continuous	0.79 (0.65,0.96)	0.016	0.77 (0.63,0.93)	0.008	0.25 (0.18,0.36)	<0.001
		T1	Ref		Ref		Ref	
		T2	0.91 (0.83,0.99)	0.031	0.90 (0.82,0.98)	0.016	0.82 (0.74,0.90)	<0.001
		T3	0.89 (0.82,0.97)	0.011	0.88 (0.81,0.96)	0.005	0.65 (0.57,0.74)	<0.001
	**CAR**	Continuous	1.10 (1.05,1.15)	<0.001	1.11 (1.06,1.16)	<0.001	1.13 (1.07,1.18)	<0.001
		T1	Ref		Ref		Ref	
		T2	1.25 (1.14,1.36)	<0.001	1.24 (1.13,1.36)	<0.001	1.16 (1.04,1.30)	0.011
		T3	1.37 (1.26,1.50)	<0.001	1.39 (1.27,1.52)	<0.001	1.40 (1.24,1.58)	<0.001
	**HRR and CAR**	High HRR and Low CAR	Ref		Ref		Ref	
		Low HRR and Low CAR	1.14 (0.99,1.31)	0.077	1.15 (1.00,1.32)	0.055	1.37 (1.17,1.60)	<0.001
		High HRR and High CAR	1.29 (1.18,1.41)	<0.001	1.30 (1.19,1.42)	<0.001	1.27 (1.15,1.40)	<0.001
		Low HRR and High CAR	1.45 (1.31,1.61)	<0.001	1.47 (1.33,1.63)	<0.001	1.73 (1.52,1.97)	<0.001
**MIMIC-III**	**HRR**	Continuous	0.42 (0.32,0.55)	<0.001	0.41 (0.31,0.53)	<0.001	0.12 (0.08,0.20)	<0.001
		T1	Ref		Ref		Ref	
		T2	0.78(0.70,0.87)	<0.001	0.77 (0.69,0.86)	<0.001	0.67 (0.60,0.76)	<0.001
		T3	0.70(0.62,0.78)	<0.001	0.69 (0.61,0.77)	<0.001	0.52 (0.45,0.61)	<0.001
	**CAR**	Continuous	1.17 (1.09,1.25)	<0.001	1.18 (1.10,1.26)	<0.001	1.13 (1.05,1.22)	0.001
		T1	Ref		Ref		Ref	
		T2	1.24 (1.10,1.38)	<0.001	1.23 (1.10,1.38)	<0.001	1.15 (0.99,1.33)	0.059
		T3	1.48 (1.32,1.65)	<0.001	1.48 (1.33,1.66)	<0.001	1.27 (1.09,1.47)	0.002
	**HRR and CAR**	High HRR and Low CAR	Ref		Ref		Ref	
		Low HRR and Low CAR	1.54 (1.27,1.86)	<0.001	1.57 (1.29,1.90)	<0.001	1.72 (1.38,2.13)	<0.001
		High HRR and High CAR	1.35 (1.22,1.49)	<0.001	1.36 (1.23,1.50)	<0.001	1.22 (1.10,1.36)	<0.001
		Low HRR and High CAR	1.70 (1.45,1.99)	<0.001	1.71 (1.46,2.00)	<0.001	1.59 (1.33,1.92)	<0.001

**eICU-CRD**	**HRR**	Continuous	0.39 (0.32,0.48)	<0.001	0.44 (0.35,0.54)	<0.001	1.32 (0.93,1.87)	0.126
		T1	Ref		Ref		Ref	
		T2	0.77 (0.70,0.84)	<0.001	0.77 (0.71,0.85)	<0.001	1.03 (0.93,1.14)	0.565
		T3	0.69 (0.63,0.76)	<0.001	0.74 (0.67,0.81)	<0.001	1.12 (0.97,1.30)	0.113
	**CAR**	Continuous	1.07 (1.05,1.08)	<0.001	1.07 (1.06,1.09)	<0.001	1.09 (1.06,1.11)	<0.001
		T1	Ref		Ref		Ref	
		T2	1.71 (1.51,1.93)	<0.001	1.70 (1.50,1.92)	<0.001	1.59 (1.39,1.81)	<0.001
		T3	3.12 (2.79,3.49)	<0.001	3.20 (2.85,3.59)	<0.001	2.51 (2.20,2.85)	<0.001
	**HRR and CAR**	High HRR and Low CAR	Ref		Ref		Ref	
		Low HRR and Low CAR	1.67 (1.30,2.16)	<0.001	1.62 (1.25,2.09)	<0.001	1.37 (1.06,1.78)	0.018
		High HRR and High CAR	2.56 (2.31,2.85)	<0.001	2.57 (2.30,2.86)	<0.001	2.14 (1.91,2.40)	<0.001
		Low HRR and High CAR	3.61 (3.16,4.12)	<0.001	3.58 (3.14,4.09)	<0.001	2.31 (1.98,2.70)	<0.001

Model 1: Adjusted only for the main exposure variable (HRR, CAR, or joint group).

Model 2: Model 1 plus age, gender and weight.

Model 3: Model 2 plus comorbidities (AKI, CA, CB, CKD, HEP, MI, PNA, T2DM), laboratory parameters (ALT, AST, total bilirubin, chloride, GLU, hematocrit, platelet count, RBC, WBC, sodium), GCS, and treatments (glucocorticoid use, neuromuscular blocker use, sedative or analgesic use, ventilation).

Abbreviations: HRR, hemoglobin-to-red blood cell distribution width ratio; CAR, creatinine-to-albumin ratio; HR, hazard ratio; CI, confidence interval; Ref, reference; AKI, acute kidney injury; CA, cancer; CB, chronic bronchitis; CKD, chronic kidney disease; HEP, hepatic disease; MI, myocardial infarction; PNA, pneumonia; T2DM, type 2 diabetes mellitus; GCS, Glasgow Coma Scale; RBC, red blood cell count; WBC, white blood cell count; ALT, alanine aminotransferase; AST, aspartate aminotransferase; GLU, glucose.

Table S6. Association of HRR and CAR with 180-Day Mortality in Three critical ill Cohorts

Cohort	Exposure	Level	Model 1 HR (95% CI)	*p* value	Model 2 HR (95% CI)	*p* value	Model 3 HR (95% CI)	*p* value
**MIMIC-IV**	**HRR**	Continuous	0.72 (0.60,0.87)	0.001	0.70 (0.58,0.85)	<0.001	0.23 (0.17,0.32)	<0.001
		T1	Ref		Ref		Ref	
		T2	0.90 (0.83,0.97)	0.009	0.89 (0.82,0.96)	0.004	0.80 (0.73,0.88)	<0.001
		T3	0.85 (0.78,0.92)	<0.001	0.84 (0.77,0.91)	<0.001	0.62 (0.54,0.70)	<0.001
	**CAR**	Continuous	1.09 (1.05,1.14)	<0.001	1.11 (1.06,1.16)	<0.001	1.11 (1.06,1.17)	<0.001
		T1	Ref		Ref		Ref	
		T2	1.23 (1.13,1.34)	<0.001	1.23 (1.13,1.33)	<0.001	1.14 (1.04,1.26)	0.004
		T3	1.36 (1.25,1.47)	<0.001	1.37 (1.26,1.49)	<0.001	1.32 (1.20,1.45)	<0.001
	**HRR and CAR**	High HRR and Low CAR	Ref		Ref		Ref	
		Low HRR and Low CAR	1.13 (0.99,1.29)	0.066	1.14 (1.00,1.31)	0.047	1.35 (1.16,1.56)	<0.001
		High HRR and High CAR	1.26 (1.16,1.37)	<0.001	1.27 (1.17,1.38)	<0.001	1.24 (1.13,1.36)	<0.001
		Low HRR and High CAR	1.47 (1.33,1.61)	<0.001	1.49 (1.35,1.64)	<0.001	1.71 (1.51,1.93)	<0.001
**MIMIC-III**	**HRR**	Continuous	0.34 (0.27,0.44)	<0.001	0.33 (0.26,0.42)	<0.001	0.09 (0.06,0.14)	<0.001
		T1	Ref		Ref		Ref	
		T2	0.77 (0.69,0.84)	<0.001	0.76 (0.68,0.83)	<0.001	0.66 (0.60,0.74)	<0.001
		T3	0.65 (0.59,0.72)	<0.001	0.64 (0.58,0.71)	<0.001	0.49 (0.43,0.57)	<0.001
	**CAR**	Continuous	1.18 (1.11,1.25)	<0.001	1.19 (1.12,1.26)	<0.001	1.15 (1.08,1.23)	<0.001
		T1	Ref		Ref		Ref	
		T2	1.25 (1.13,1.39)	<0.001	1.25 (1.12,1.38)	<0.001	1.18 (1.06,1.32)	0.003
		T3	1.52 (1.38,1.69)	<0.001	1.53 (1.38,1.70)	<0.001	1.36 (1.21,1.53)	<0.001
	**HRR and CAR**	High HRR and Low CAR	Ref		Ref		Ref	
		Low HRR and Low CAR	1.57 (1.32,1.88)	<0.001	1.61 (1.35,1.93)	<0.001	1.75 (1.44,2.13)	<0.001
		High HRR and High CAR	1.38 (1.26,1.50)	<0.001	1.38 (1.26,1.51)	<0.001	1.26 (1.14,1.40)	<0.001
		Low HRR and High CAR	1.83 (1.58,2.11)	<0.001	1.84 (1.59,2.12)	<0.001	1.69 (1.43,2.00)	<0.001

**eICU-CRD**	**HRR**	Continuous	0.39 (0.32,0.48)	<0.001	0.44 (0.35,0.54)	<0.001	1.31 (0.92,1.86)	0.131
		T1	Ref		Ref		Ref	
		T2	0.77 (0.71,0.84)	<0.001	0.77 (0.71,0.85)	<0.001	1.03 (0.93,1.14)	0.560
		T3	0.69 (0.63,0.76)	<0.001	0.74 (0.67,0.81)	<0.001	1.12 (0.97,1.30)	0.114
	**CAR**	Continuous	1.07 (1.05,1.08)	<0.001	1.07 (1.06,1.09)	<0.001	1.09 (1.06,1.11)	<0.001
		T1	Ref		Ref		Ref	
		T2	1.71 (1.51,1.93)	<0.001	1.70 (1.50,1.92)	<0.001	1.58 (1.39,1.80)	<0.001
		T3	3.12 (2.79,3.49)	<0.001	3.20 (2.85,3.59)	<0.001	2.45 (2.16,2.78)	<0.001
	**HRR and CAR**	High HRR and Low CAR	Ref		Ref		Ref	
		Low HRR and Low CAR	1.67 (1.30,2.16)	<0.001	1.62 (1.25,2.09)	<0.001	1.37 (1.06,1.78)	0.018
		High HRR and High CAR	2.56 (2.31,2.85)	<0.001	2.57 (2.30,2.86)	<0.001	2.14 (1.91,2.40)	<0.001
		Low HRR and High CAR	3.61 (3.17,4.12)	<0.001	3.59 (3.14,4.10)	<0.001	2.32 (1.99,2.70)	<0.001

Model 1: Adjusted only for the main exposure variable (HRR, CAR, or joint group).

Model 2: Model 1 plus age, gender and weight.

Model 3: Model 2 plus comorbidities (AKI, CA, CB, CKD, HEP, MI, PNA, T2DM), laboratory parameters (ALT, AST, total bilirubin, chloride, GLU, hematocrit, platelet count, RBC, WBC, sodium), GCS, and treatments (glucocorticoid use, neuromsuscular blocker use, sedative or analgesic use, ventilation).

Abbreviations: HRR, hemoglobin-to-red blood cell distribution width ratio; CAR, creatinine-to-albumin ratio; HR, hazard ratio; CI, confidence interval; Ref, reference; AKI, acute kidney injury; CA, cancer; CB, chronic bronchitis; CKD, chronic kidney disease; HEP, hepatic disease; MI, myocardial infarction; PNA, pneumonia; T2DM, type 2 diabetes mellitus; GCS, Glasgow Coma Scale; RBC, red blood cell count; WBC, white blood cell count; ALT, alanine aminotransferase; AST, aspartate aminotransferase; GLU, glucose.

Table S7. Association of HRR and CAR with 1-Year Mortality in Three critical ill Cohorts

Cohort	Exposure	Level	Model 1 HR (95% CI)	*p* value	Model 2 HR (95% CI)	*p* value	Model 3 HR (95% CI)	*p* value
**MIMIC-IV**	**HRR**	Continuous	0.70 (0.59,0.83)	<0.001	0.69 (0.58,0.82)	<0.001	0.23 (0.17,0.32)	<0.001
		T1	Ref		Ref		Ref	
		T2	0.90 (0.83,0.97)	0.007	0.89 (0.83,0.96)	0.004	0.82 (0.75,0.89)	<0.001
		T3	0.85 (0.79,0.92)	<0.001	0.85 (0.78,0.91)	<0.001	0.64 (0.57,0.72)	<0.001
	**CAR**	Continuous	1.10 (1.06,1.14)	<0.001	1.11 (1.07,1.16)	<0.001	1.11 (1.06,1.16)	<0.001
		T1	Ref		Ref		Ref	
		T2	1.19 (1.10,1.29)	<0.001	1.19 (1.10,1.28)	<0.001	1.11 (1.02,1.21)	0.015
		T3	1.33 (1.23,1.44)	<0.001	1.34 (1.24,1.45)	<0.001	1.28 (1.17,1.40)	<0.001
	**HRR and CAR**	High HRR and Low CAR	Ref		Ref		Ref	
		Low HRR and Low CAR	1.12 (0.99,1.27)	0.064	1.13 (1.00,1.28)	0.047	1.32 (1.15,1.51)	<0.001
		High HRR and High CAR	1.23 (1.14,1.33)	<0.001	1.23 (1.14,1.33)	<0.001	1.20 (1.10,1.31)	<0.001
		Low HRR and High CAR	1.43 (1.31,1.57)	<0.001	1.45 (1.33,1.59)	<0.001	1.63 (1.45,1.83)	<0.001
**MIMIC-III**	**HRR**	Continuous	0.31 (0.25,0.39)	<0.001	0.30 (0.24,0.38)	<0.001	0.08 (0.06,0.12)	<0.001
		T1	Ref		Ref		Ref	
		T2	0.74 (0.67,0.81)	<0.001	0.73 (0.66,0.80)	<0.001	0.64 (0.58,0.71)	<0.001
		T3	0.63 (0.58,0.70)	<0.001	0.62 (0.57,0.68)	<0.001	0.48 (0.42,0.55)	<0.001
	**CAR**	Continuous	1.20 (1.13,1.26)	<0.001	1.20 (1.14,1.27)	<0.001	1.18 (1.11,1.25)	<0.001
		T1	Ref		Ref		Ref	
		T2	1.23 (1.12,1.36)	<0.001	1.23 (1.11,1.35)	<0.001	1.18 (1.06,1.31)	0.002
		T3	1.52 (1.38,1.66)	<0.001	1.53 (1.39,1.68)	<0.001	1.39 (1.25,1.55)	<0.001
	**HRR and CAR**	High HRR and Low CAR	Ref		Ref		Ref	
		Low HRR and Low CAR	1.61 (1.36,1.90)	<0.001	1.65 (1.39,1.95)	<0.001	1.78 (1.48,2.14)	<0.001
		High HRR and High CAR	1.40 (1.28,1.52)	<0.001	1.41 (1.29,1.53)	<0.001	1.32 (1.20,1.44)	<0.001
		Low HRR and High CAR	1.87 (1.63,2.15)	<0.001	1.89 (1.64,2.16)	<0.001	1.76 (1.50,2.07)	<0.001

**eICU-CRD**	**HRR**	Continuous	0.39 (0.32,0.48)	<0.001	0.44 (0.35,0.54)	<0.001	1.31 (0.92,1.86)	0.131
		T1	Ref		Ref		Ref	
		T2	0.77 (0.71,0.84)	<0.001	0.77 (0.71,0.85)	<0.001	1.03 (0.93,1.14)	0.560
		T3	0.69 (0.63,0.76)	<0.001	0.74 (0.67,0.81)	<0.001	1.12 (0.97,1.30)	0.114
	**CAR**	Continuous	1.07 (1.05,1.08)	<0.001	1.07 (1.06,1.09)	<0.001	1.09 (1.06,1.11)	<0.001
		T1	Ref		Ref		Ref	
		T2	1.71 (1.51,1.93)	<0.001	1.70 (1.50,1.92)	<0.001	1.58 (1.39,1.80)	<0.001
		T3	3.12 (2.79,3.49)	<0.001	3.20 (2.85,3.59)	<0.001	2.45 (2.16,2.78)	<0.001
	**HRR and CAR**	High HRR and Low CAR	Ref		Ref		Ref	
		Low HRR and Low CAR	1.67 (1.30,2.16)	<0.001	1.62 (1.25,2.09)	<0.001	1.37 (1.06,1.78)	0.018
		High HRR and High CAR	2.56 (2.31,2.85)	<0.001	2.57 (2.30,2.86)	<0.001	2.14 (1.91,2.40)	<0.001
		Low HRR and High CAR	3.61 (3.17,4.12)	<0.001	3.59 (3.14,4.10)	<0.001	2.32 (1.99,2.70)	<0.001

Model 1: Adjusted only for the main exposure variable (HRR, CAR, or joint group).

Model 2: Model 1 plus age, gender and weight.

Model 3: Model 2 plus comorbidities (AKI, CA, CB, CKD, HEP, MI, PNA, T2DM), laboratory parameters (ALT, AST, total bilirubin, chloride, GLU, hematocrit, platelet count, RBC, WBC, sodium), GCS, and treatments (glucocorticoid use, neuromuscular blocker use, sedative or analgesic use, ventilation).

Abbreviations: HRR, hemoglobin-to-red blood cell distribution width ratio; CAR, creatinine-to-albumin ratio; HR, hazard ratio; CI, confidence interval; Ref, reference; AKI, acute kidney injury; CA, cancer; CB, chronic bronchitis; CKD, chronic kidney disease; HEP, hepatic disease; MI, myocardial infarction; PNA, pneumonia; T2DM, type 2 diabetes mellitus; GCS, Glasgow Coma Scale; RBC, red blood cell count; WBC, white blood cell count; ALT, alanine aminotransferase; AST, aspartate aminotransferase; GLU, glucose.

Table S9. Comparison of AUC for Joint Analysis, SOFA, and SAPSII across Different Time Points with DeLong Test

Dataset	Outcome	Joint AUC	SOFA AUC	△AUC	*p* value	SAPSII AUC	△AUC	*p* value
MIMIC IV	28days	0.684	0.689	-0.005	0.312	0.693	-0.009	0.072
	90days	0.687	0.682	0.005	0.261	0.690	-0.003	0.566
	180days	0.688	0.676	0.012	0.021	0.688	0.000	0.989
	1year	0.680	0.656	0.025	0.001	0.673	0.008	0.353
MIMIC III	28days	0.671	0.676	-0.005	0.410	0.693	-0.022	0.002
	90days	0.679	0.674	0.005	0.442	0.691	-0.013	0.065
	180days	0.701	0.683	0.017	0.006	0.700	0.001	0.857
	1year	0.705	0.676	0.029	0.000	0.695	0.010	0.229
eicu-CRD	28days	0.736	0.729	0.007	0.002	0.739	-0.003	0.195
	90days	0.730	0.724	0.006	0.005	0.733	-0.003	0.164
	180days	0.730	0.724	0.006	0.004	0.733	-0.003	0.176
	1year	0.730	0.724	0.006	0.004	0.733	-0.003	0.176

Notes: The DeLong test was performed to compare the AUC differences (△AUC) between the models, and *p* values are provided to assess the statistical significance of the differences.

Abbreviations: AUC: Area Under the Curve; Joint: A four-level variable derived from HRR and CAR levels; SOFA: Sequential Organ Failure Assessment; SAPSII: Simplified Acute Physiology Score II; △AUC: Difference in AUC between the joint analysis and individual models; MIMIC-IV: Medical Information Mart for Intensive Care IV; MIMIC-III: Medical Information Mart for Intensive Care III; eICU-CRD: eICU Collaborative Research Database.

Table S10. Variance Inflation Factor of Each Variable for Predicting the Primary Outcome in the MIMIC-III, MIMIC-IV, and EICU-CRD Databases

Variable Name	VIF for MIMIC-III 28-Day Outcome			VIF for MIMIC-IV 28-Day Outcome			VIF for EICU-CRD 28-Day Outcome		
	HRR	CAR	Joint	HRR	CAR	Joint	HRR	CAR	Joint
**Age**	1.071	1.070	1.070	1.148	1.156	1.148	1.068	1.067	1.073
**Gender**	1.074	1.080	1.087	1.055	1.058	1.060	1.042	1.041	1.040
**Weight**	1.119	1.120	1.121	1.130	1.124	1.130	1.071	1.071	1.075
**AKI**	1.070	1.076	1.117	1.064	1.067	1.093	1.053	1.054	1.072
**HEP**	1.028	1.029	1.032	1.080	1.092	1.092	1.046	1.046	1.048
**PNA**	1.045	1.046	1.047	1.054	1.053	1.054	1.082	1.080	1.080
**CKD**	1.059	1.060	1.074	1.094	1.113	1.138	1.069	1.064	1.067
**CA**	1.015	1.016	1.018	1.027	1.027	1.027	1.062	1.060	1.058
**T2DM**	1.060	1.062	1.064	1.093	1.096	1.093	1.029	1.028	1.028
**CB**	1.041	1.040	1.042	1.029	1.030	1.030	1.002	1.002	1.002
**MI**	1.040	1.029	1.030	1.026	1.026	1.024	1.037	1.036	1.036
**Hematocrit**	2.475	2.150	2.241	3.123	2.561	2.733	2.621	2.197	2.267
**Platelet count**	1.055	1.052	1.054	1.053	1.053	1.053	1.077	1.076	1.078
**RBC**	2.124	2.178	2.207	2.584	2.575	2.616	2.223	2.205	2.206
**WBC**	1.017	1.017	1.030	1.033	1.027	1.034	1.052	1.045	1.044
**Chloride**	1.533	1.538	1.533	1.538	1.545	1.536	1.589	1.592	1.585
**Glu**	1.055	1.050	1.052	1.086	1.082	1.084	1.039	1.039	1.040
**Sodium**	1.510	1.509	1.512	1.531	1.519	1.518	1.585	1.581	1.576
**ALT**	2.830	2.808	2.808	2.024	2.047	2.024	1.385	1.382	1.386
**AST**	2.811	2.792	2.790	2.021	2.045	2.019	1.366	1.361	1.362
**Bilirubin total**	1.101	1.090	1.097	1.128	1.135	1.137	1.144	1.106	1.128

**HRR/CAR/Joint**	1.686	1.040	1.103	1.806	1.090	1.130	1.720	1.037	1.081
**GCS**	1.013	1.013	1.013	1.010	1.010	1.011	1.015	1.015	1.015
**Ventilation**	1.216	1.215	1.214	1.044	1.046	1.044	1.120	1.119	1.120
**Glucocorticoid**	1.062	1.058	1.060	1.054	1.053	1.053	1.074	1.073	1.074
**Neuromuscular blocker**	1.011	1.013	1.013	1.036	1.031	1.032	1.020	1.020	1.019
**Sedative analgesic**	1.221	1.224	1.222	1.095	1.096	1.094	1.113	1.113	1.110

Abbreviations: AKI, Acute Kidney Injury; HEP, Hepatitis; PNA, Pneumonia; CKD, Chronic Kidney Disease; CA, Cancer; T2DM, Type 2 Diabetes Mellitus; CB, Chronic Bronchitis; MI, Myocardial Infarction; RBC, Red Blood Cell count; WBC, White Blood Cell count; Glu, Glucose; ALT, Alanine Aminotransferase; AST, Aspartate Aminotransferase; CAR, Creatinine to Albumin Ratio; HRR, Hemoglobin to Red Cell Distribution Width ratio; GCS, Glasgow Coma Scale.
